# Anti-inflammatory effects of natural polysaccharides: molecular mechanisms and nanotherapeutic applications

**DOI:** 10.3389/fimmu.2025.1723346

**Published:** 2025-12-15

**Authors:** Jihao Yang, Kai Xiong, Tengzhen Li, Morang Zhang, Zhangyun Li, Zhili Wen, Yuchuan Jiang

**Affiliations:** 1 Department of Gastroenterology, The Second Affiliated Hospital, Jiangxi Medical College, Nanchang University, Jiangxi, China; 2 School of Acupuncture and Tuina, Guizhou University of Traditional Chinese Medicine, Guiyang, China

**Keywords:** natural polysaccharides, anti-inflammation, molecular mechanism, nanonization, therapeutic application

## Abstract

Chronic excessive inflammation drives the pathogenesis of diseases such as Heart Failure (HF) and arthritis. Natural polysaccharides, with low toxicity and biodegradability, exert anti-inflammatory effects by regulating core inflammatory signaling pathways (e.g., Nuclear Factor-κB (NF-κB), Mitogen-Activated Protein Kinase (MAPK), Toll-Like Receptor (TLR)) and downregulating pro-inflammatory cytokines including Tumor Necrosis Factor-α (TNF-α), IL-1β, and IL-6. But their poor water solubility and easy breakdown by digestive enzymes limit bioavailability. Nanonization solves these problems by enhancing aqueous dispersibility, reducing enzymatic hydrolysis, and improving targeting efficiency (passive via the Enhanced Permeability and Retention (EPR) effect, active via ligand modification). It also strengthens the inhibition of pro-inflammatory pathways, activates the Nuclear Factor Erythroid 2-Related Factor 2 (Nrf2)/Heme Oxygenase-1 (HO-1) antioxidant pathway, and protects the mucosal barrier. This review is divided into four logical sections—fundamental mechanisms of inflammation and polysaccharide regulation, anti-inflammatory activities of natural polysaccharides, nanonization strategies for efficacy enhancement, and clinical translation potential. It eliminates redundancy, integrates overlapping information, and provides a concise framework to promote the clinical application of polysaccharide-based anti-inflammatory therapies.

## 1 Introduction

Inflammation is a defensive response of the body to infection or injury, but chronic and excessive inflammation leads to pathological states. It has both protective roles (eliminating pathogens and repairing tissues) and pathogenic effects (e.g., inducing Heart Failure (HF) and arthritis) (1, 2). The core mechanism includes immune response dysregulation (e.g., excessive release of TNF-α, IL-1β, IL-6), oxidative stress, and tissue damage, which may develop into multiple organ failure (e.g., sepsis) (3–5). Conventional anti-inflammatory therapies, including Non-Steroidal Anti-Inflammatory Drugs (NSAIDs), corticosteroids, biologics (e.g., anti-TNF-α antibodies), and antibiotics, have inherent limitations: they relieve symptoms in the short term but do not cure the root cause, may cause drug resistance (e.g., ineffective anti-TNF-α antibodies), have safety risks (e.g., osteoporosis from long-term corticosteroid use), lack good targeting (easily damaging healthy tissues), and have low bioavailability (susceptible to degradation and hard to penetrate biological barriers) (6–9). Natural polysaccharides, widely present in plants, fungi, and marine organisms, are promising alternatives because of their low toxicity, biodegradability, and bioactivities such as antioxidant and anti-inflammatory effects (10, 11). Their anti-inflammatory effects are achieved by regulating signaling pathways, immune cell polarization, and inflammatory cytokine expression, but poor water solubility and easy breakdown by digestive enzymes restrict their clinical efficacy (12). Unlike other compounds that often focus on a single mechanism (such as blocking inflammatory factors), natural polysaccharides exert synergistic anti-inflammatory effects through gut microbiota regulation (as prebiotics) and multi-pathway inhibition (such as antioxidant and immune regulation) (13, 14). Also, the macromolecular properties of polysaccharides make them easier to use in drug delivery systems (such as nanoparticles), improving therapeutic efficiency (15–17). Nanonization technology has become a key strategy to optimize these properties. Combining polysaccharides with traditional anti-inflammatory drugs or probiotics further forms a synergistic anti-inflammatory system. To provide a comprehensive and concise understanding of this field, this review first clarifies the fundamental mechanisms of inflammation and how polysaccharides regulate these processes, then details the anti-inflammatory activities of natural polysaccharides (closely linked to their molecular structures), explains how nanonization enhances their efficacy, and finally analyzes their clinical translation potential and challenges. It builds a logical chain from basic research to application without redundant subsections.

## 2 Fundamental mechanisms: inflammation regulation and polysaccharide targets

### 2.1 Core driving mechanisms of inflammation occurrence

Inflammation is driven by the interaction of signaling molecules, immune cells, and intracellular regulatory networks. Signaling molecules such as Specialized Pro-Resolving Mediators (SPMs)—derived from polyunsaturated fatty acids—initiate resolution signals in acute inflammation. They downregulate IL-1β and TNF-α to stop the progression to chronic inflammation. Pro-inflammatory mediators like Neutrophil Extracellular Traps (NETs) promote chronic inflammation when their levels are imbalanced (18–22). Dysregulation of these molecules leads to mediator imbalance and the development of chronic inflammatory diseases (23). Immune cells, especially macrophages, have high plasticity and a “polarization spectrum” (M1 pro-inflammatory/M2 anti-inflammatory) regulated by microenvironmental signals. M1 macrophages, activated by stimuli such as Lipopolysaccharide (LPS) and Interferon-γ (IFN-γ), secrete IL-1β, IL-6, and TNF-α to worsen inflammation and cause tissue damage. M2 macrophages, induced by IL-4 and IL-10, secrete IL-10 and Transforming Growth Factor-β (TGF-β) to promote inflammation resolution, phagocytosis, and tissue regeneration (24–29). Metabolic reprogramming (e.g., glycolysis for M1 polarization, oxidative phosphorylation for M2 polarization) and epigenetic modifications further regulate this process (30–32). Also, the interaction between autophagy and inflammasomes plays a key role. Autophagy inhibits the activation of the NOD-Like Receptor Pyrin Domain-Containing 3 (NLRP3) inflammasome by degrading damaged mitochondria (reducing the release of Reactive Oxygen Species (ROS) and Damage-Associated Molecular Patterns (DAMPs)). It also directly clears inflammasome components (e.g., the adapter protein ASC via the autophagy receptor p62) and inhibits the maturation and secretion of IL-1β and IL-18 (33–36). On the other hand, abnormal activation of the NLRP3 inhibits autophagy via IL-1β and disrupts immunometabolism (e.g., lipid and amino acid metabolic pathways), forming a positive feedback loop that aggravates inflammatory damage (37–39).

### 2.2 Key signaling pathways regulated by polysaccharides in inflammation

Natural polysaccharides regulate inflammatory responses by targeting core inflammatory signaling pathways, and their regulatory effects depend on structural characteristics. As the core pathway of inflammatory regulation, the NF-κB pathway is either inhibited or activated by polysaccharides. For example, plant polysaccharides regulate the TLR4/NF-κB axis to reduce the levels of inflammatory mediators such as Nitric Oxide (NO) and TNF-α. Sulfated polysaccharides can activate the MAPK/Akt/NF-κB pathway in RAW264.7 macrophages to promote cell proliferation and cytokine secretion. Molecular Weight (MW) and Degree of Sulfation (DS) directly affect the binding affinity of polysaccharides to receptors like TLR, thus determining whether the NF-κB pathway is activated or inhibited (40–45). But this argument does not clarify the core mechanistic differences in the bidirectional regulation of the NF-κB pathway by different polysaccharides. The activation and inhibition effects of the same pathway may come from differences in the fine structures of polysaccharides, such as the type of glycosidic bonds and the degree of sulfation. The cyclic Adenosine Monophosphate (cAMP) pathway, a key second messenger system, interacts closely with the NF-κB pathway in the anti-inflammatory process. When the NF-κB pathway upregulates Phosphodiesterase 4 (PDE4) to accelerate cAMP degradation and sustain inflammation, polysaccharides can interfere with this process (e.g., by inhibiting PDE4) to increase cAMP levels, thereby indirectly inhibiting inflammatory responses. Some polysaccharides also target the GPR91-Gαi-cAMP-NF-κB pathway to promote cell apoptosis in fibrosis models (46, 47). The MAPK pathway, which includes three main subtypes (p38 MAPK, c-Jun N-Terminal Kinase (JNK), Extracellular Signal-Regulated Kinase (ERK)) and is closely related to pro-inflammatory factor translation, cell apoptosis, and oxidative stress, is regulated by polysaccharides mainly through selective inhibition of pro-inflammatory subtypes (p38 MAPK and JNK).

In cell models stimulated by oxidative stress or LPS, polysaccharides reduce the gene transcription of Matrix Metalloproteinase-9 (MMP-9) and cell invasion by inhibiting JNK and p38 activation (relying on Myc-mediated transcriptional regulation). They also decrease the phosphorylation levels of ERK, JNK, and p38 to reduce the expression of pro-inflammatory cytokines such as TNF-α and IL-6, or upregulate Dual-Specificity Phosphatase 1 (DUSP1) to inhibit MAPK activation and alleviate neuroinflammation. They can also interfere with inflammation through the TLR4/MAPK crosstalk pathway, such as inhibiting the activation of the TLR4/MAPK pathway to reduce the expression of NO, IL-6, and TNF-α (48–51). The TLR pathway, a pattern recognition receptor pathway in innate immunity, is bidirectionally regulated by polysaccharides. Polysaccharides can block LPS-induced inflammation by downregulating TLR4 expression or inhibiting its activation (e.g., reducing TLR4-mediated signal transduction through interaction with TLR4, or inhibiting the activation of the TLR4/MAPK pathway by preventing TLR4 dimerization and downstream molecule recruitment). They also bind to TLRs (e.g., TLR2, TLR4) to initiate adaptive immune responses, activate immune signals in macrophages and dendritic cells, and promote the secretion of anti-inflammatory factors such as IL-10 (52–54). Also, the Phosphatidylinositol 3-Kinase (PI3K)/Akt pathway— involved in cell survival, metabolism, and immune responses—is targeted by polysaccharides to reduce the phosphorylation levels of PI3K and Akt, directly cutting off downstream pro-inflammatory signals (e.g., NF-κB, MAPK). The Janus Kinase (JAK)/Signal Transducer and Activator of Transcription (STAT) pathway (a core cytokine-mediated inflammatory pathway) is regulated by polysaccharides to block the activation of JAK kinases (e.g., JAK2) and the phosphorylation of STAT3. This prevents the transcription of pro-inflammatory genes and promotes the secretion of anti-inflammatory factors such as IL-10 to balance inflammatory responses (55–58).

## 3 Anti-inflammatory activities of natural polysaccharides

### 3.1 Classification, structure, and activity specificity

Natural polysaccharides with anti-inflammatory activity are mainly derived from three categories, each with unique structural characteristics that determine their anti-inflammatory specificity. Plant-derived polysaccharides include those from *Moringa oleifera* seeds (e.g., MOSP-1), bamboo shoots, common buckwheat (*Fagopyrum esculentum* Moench, FEP), and *Nitraria tangutorum* Bobr. (NTP). They regulate inflammatory responses through mechanisms such as inhibiting the release of pro-inflammatory cytokines, scavenging ROS, or modulating gut microbiota (59–63). Fungal-derived polysaccharides—such as sulfated polysaccharides from *Poria cocos*, polysaccharides from *Dictyophora indusiata*, *Pleurotus eryngii*, and *Flammulina velutipes*—inhibit the production of NO and the activation of the NF-κB pathway to exert anti-inflammatory effects (64–67). Marine polysaccharides, including jellyfish skin polysaccharides (JSP) and sulfated polysaccharides extracted from various seafood (algae, marine animals), exhibit anti-inflammatory, antioxidant, and immunomodulatory activities in colitis models and alleviate obesity-related inflammation (68, 69). Other sources of anti-inflammatory polysaccharides include algae (unspecified species) and pitaya (dragon fruit) stems and peels, which also show potential for treating inflammatory injuries (70, 71). The anti-inflammatory specificity of natural polysaccharides is determined by their molecular structures (**Table 1**). Monosaccharide composition regulates the selection of anti-inflammatory pathways. *Ganoderma lucidum* polysaccharides, which have a β-(1→3)-glucan main chain and β-(1→6)-glucose side chain branches, can specifically bind to the Dectin-1 receptor on macrophages, activate M2 polarization, and inhibit the NF-κB pathway to reduce the release of pro-inflammatory cytokines (97, 98). The glucose/galactose ratio in monosaccharides affects receptor recognition—a high glucose ratio enhances the binding affinity to Dectin-1, while galactose residues may inhibit NF-κB activation by regulating TLR4 endocytosis (99).

**Table 1:** Chemical composition of polysaccharides.

Name	Source	MW	Composition and content	Types	References
Low MW Fucoidan (LMF)	Brown algae *Undaria pinnatifida* (Clinical grade)	4.4 g × 2/day oral preparation→1–5 kDa	Fuc > 90, containing sulfate ester	Mainly α-(1→3)/(1→4)-L-FucpS	(72)
GLP	Red algae *Gracilaria lemaneiformis*	2.305 × 10^6^ Da	Gal 93.65, Xyl 3.49, Glc 2.86	Alternating β-(1→3)/α-(1→4) galactose	(73)
PS-HBS	Herb *Gymnopetalum cochinchinense*	1.217 × 10^5^ Da	Glc + Fru (repeating unit)	(1→6)-Glcp, (2→6)/(2→4)-Fruf	(74)
Pectin with specific structure	Passion fruit peel	20–500 kDa (fraction)	GalA main, Rha secondary	HG: α-(1→4)-GalA; RG-I: →2,4-α-Rhap→	(75)
TOP60-1	Fungus *Trametes orientalis*	1.122 × 10^4^ Da	Glc, Gal, Man, Fuc	→4/3/6-β-Glcp and →2,6-α-Galp, etc. with high branching	(76)
PEP-A/B/C	*Phyllanthus emblica* fruit	Multimodal: 8–310 kDa	Ara, Gal, Glc, Rha	β-D-Glcp backbone, multiple substitutions on side chains	(77)
DXBP-0	Fungus *Dictyophora rubrovolvata*	2.711 × 10³ kDa	Glc 100	→3)-β-Glcp.(1→ backbone, →3,6-β-Glcp branches	(78)
Nacre-PS	Nacreous layer	~5 kDa	Glc + Man + Rha account for 87	Not detailed (neutral glycosidic linkages)	(79)
PSH	Fungus *Sanghuangporus vaninii*	5.25 × 10^4^ Da	Glc, Gal, Ara	Glcp-(1→, →4-Glcp, →3-Galp, Araf-(1→	(80)
DNJP	*Morinda citrifolia* juice	1.918 × 10^5^ Da	Glc, Gal, Ara, Man	Original glycosidic linkages retained, Mw↓ after depolymerization	(81)
LP	Bulb of *Lilium brownii*	2.4006 × 10^4^ Da	Glc: Man = 1:1.56	Not clear; presumed α-(1→4)/β-(1→4)	(82)
Medium MW AX	*Plantago asiatica* medium Mw Arabinoxylan	Approximately 30–50 kDa	Xyl main, Ara branches	β-(1→4)-Xylp backbone, O-3-Ara branches	(83)
EKPA	*Epimedium koreanum*	1.258 × 10^5^ Da	Glc, GlcA, Gal	1,4-α-Glcp/GlcAp backbone, 1,3,6-β-Galp branches	(84)
NCP-DES-3	Cyanobacteria *Nostoc commune*	7.31% extract; Mw ≈ 1–2 × 10^5^ Da	Rha, GalA, Glc, Xyl, etc.	→3)-β-Glcp,(1→ with multiple branches	(85)
BRL-G	Root of *Brassica rapa*	Initial ~450 kDa, <200 kDa after fermentation	FucT up to 25.6, XylT up to 26.9	RG-I/AX complex; containing →6-Gal, →4-Glc	(86)
DSA (Formaldehyde-free DAP)	Sodium alginate periodate oxidation	Mw not given, polyaldehyde modification	Man-β-(1→4)-uronic backbone	Retaining β-(1→4) after Dialdehyde modification	(87)
SZ	Leech *Hirudo nipponica*	2.2128 × 10^5^ Da	Glc main	→4-α-Glcp-(1→; O-3/O-6 branches	(88)
IRPS-TE-3	Traditional Chinese medicine *Isatidis Radix*	~1.6 × 10³ Da	GalA, Araf, Gal	Backbone →4-α-GalpA-(1/→5-α-Araf-(1→	(89)
CDPS-1	*Cistanche deserticola*	989 Da	Polysaccharide + phenol 5.94	Oligomeric: presumed β-(1→4)/α-(1→6)	(90)
PKP1	*Polygonatum kingianum*	5.3 × 10³ Da	Fru & Glc	β-D-Fruf-(1→2/6) + α-Glcp-(1→6) backbone	(91)
O-antigen PS	*Salmonella Paratyphi* A	Medium Mw retained >90 kDa	Rha, Man, Gal, Abe, Paratose	→3)-α-Manp-(1→2)-α-Rhap- and other repeating units	(92)
PCLP	*Pholidota chinensis*	~3.8 × 10^5^ Da	Glc, Man	→4-α-Glcp-(1→4)-β-Manp-(1→ backbone	(93)
MCP-3	Herb *Mesona chinensis*	1.6014 × 10^4^ Da	GalA 29.7, Glc 20.2, Rha 17.2, Ara 7.1…	→4-α-GalpA-(1→6)/→2-α-Rhap-(1→ backbone	(94)
MPP	Mango peel pectin	6.76 × 10^5^ Da	GalA 21.36, Glc 8.85, Ara 5.97	→6-α-GalPAOMe-(1→ & →4-β-Glcp-(1→	(95)
SVP	*Sanghuang vaninii*	7.473 × 10^4^ → enzymatic degradation 5.533 × 10³ Da	Glc, Man, Gal	β-(1→3)/(1→6)-Glcp backbone	(96)

For marine sulfated polysaccharides, DS is the key to regulating the TLR4 pathway. High-DS sulfated polysaccharides (e.g., fucoidan) block the formation of the TLR4-MD2 complex through steric hindrance, thereby inhibiting MyD88-dependent NF-κB signaling. Low-DS polysaccharides tend to activate the Nrf2 pathway and upregulate the antioxidant enzyme HO-1 to alleviate inflammation (100–103). Studies on K5 sulfated polysaccharide derivatives have shown that polysaccharides with a high degree of sulfation exhibit significant anti-inflammatory activity. Among them, the K5 F2 fragment inhibits the phosphorylation of p38, while the K5 F3 fragment inhibits the activation of the p38/JNK signaling pathway, thus confirming that the degree of sulfation is the core driving factor affecting the anti-inflammatory response (104). In experiments on sulfonated carboxymethyl cellulose (CMC) derivatives, the sCMC derivative with a sulfation degree of approximately 10% showed a molecular weight of 10 kDa and conferred chondrogenic properties. It also effectively reduced key inflammatory markers in an osteoarthritis model, indicating that DS indirectly regulates anti-inflammatory mechanisms by improving the stability of polysaccharides (105). Also, Surface Plasmon Resonance (SPR) studies have shown that the binding strength between polysaccharides and growth factors mainly depends on the degree of sulfation, suggesting that DS dominates the anti-inflammatory response by regulating receptor affinity (106).

Molecular weight(MW)leads to distinct anti-inflammatory mechanisms. Low-MW polysaccharides (LMWPs, <10 kDa) such as the 5 kDa fragment of Astragalus polysaccharides (APS) can penetrate the intestinal epithelial barrier to act directly on immune cells. They inhibit excessive T cell activation by activating the PI3K/Akt pathway and upregulating the expression of immune checkpoint molecules such as TIGIT to suppress intestinal inflammatory responses (107–109). High-MW polysaccharides (HMWPs, >100 kDa) such as high-MW APS are not easily absorbed directly but can be fermented by gut microbiota to produce Short-Chain Fatty Acids (SCFAs). SCFAs inhibit Histone Deacetylase (HDAC) activity by activating the GPR43 receptor, promote the differentiation of regulatory T (Treg) cells, and block NF-κB nuclear translocation, thus exerting systemic anti-inflammatory effects (110). There is a research gap regarding the correlation between molecular weight and activity. Current studies have not addressed the activity characteristics of polysaccharides with intermediate molecular weights ranging from 10 to 100 kDa, nor have they reflected the differences in activity mechanisms of polysaccharides with the same molecular weight due to different sources.

Studies have shown that the impact of molecular weight on anti-inflammatory activity exhibits a “threshold effect” and has a synergistic interaction with DS: the combination of low molecular weight (typically <40 kDa) and high DS results in the strongest activity. Both the F2 (36 kDa) and F3 (1.9 kDa) fragments of K5 polysaccharides demonstrate excellent anti-inflammatory activity, with the highest inhibition rates of IL-6 and TNF-α production reaching 83% and 37% respectively. In contrast, the high molecular weight fragment F1 (327 kDa) lacks data supporting high activity, indicating that a molecular weight below 40 kDa is a critical threshold for optimizing anti-inflammatory activity (111). SPR experiments further confirmed that polysaccharides with molecular weights in the range of 5–40 kDa have significantly higher binding affinity to inflammatory receptors, which is associated with improved water solubility and cellular permeability. Moreover, the binding affinity mainly depends on the degree of sulfation of the polysaccharides (112). From the analysis of the dose-effect relationship, the activity differences between K5 F3 (1.9 kDa, DS 1:8) and F2 (36 kDa, DS 1:3) indicate that a decrease in molecular weight can enhance activity, even under high DS conditions. Although data on molecular weight gradients under the same DS are currently lacking, the overall findings support the conclusion that “low molecular weight combined with high DS can enhance the anti-inflammatory activity of sulfated polysaccharides” (111).

There are still gaps in current research: the anti-inflammatory activity characteristics of polysaccharides with intermediate molecular weights (10–100 kDa) have not been systematically investigated, nor has the variation in activity mechanisms of polysaccharides with the same molecular weight due to differences in sources been elucidated. The type and conformation of glycosidic bonds affect the binding of polysaccharides to anti-inflammatory receptors. The triple-helical conformation of β-(1→3)-glucans (e.g., *Grifola frondosa* polysaccharides, SPG) can form multivalent binding with the Dectin-1 receptor on macrophages, trigger the Syk/CARD9 signaling cascade, promote IL-10 secretion, and induce M2 polarization (113). Its rigid helical structure binds to receptors more stably than the flexible chain of starch α-(1→4)-glucan, resulting in more than a 3-fold increase in anti-inflammatory activity (114). α-type glycosidic bonds (e.g., →4)-α-Galp-(1→ and →4)-α-Glcp-(1→ in Jerusalem artichoke polysaccharides) can bind to the Galectin-3 receptor and inhibit the ERK/NF-κB pathway to alleviate skin inflammation. β-glycosidic bonds (e.g., →3,4)-β-GalpA-(1→) can balance immune responses by activating the TLR4/TGF-β pathway (115–117).

The configuration of glycosidic bonds indirectly regulates the binding mechanism with inflammatory receptors (such as TLRs) by influencing the conformation and charge distribution of polysaccharides. For example, (1→4) and (1→6) glycosidic bonds have been confirmed as the structural basis for immunomodulatory and anti-tumor activities, and can enhance receptor binding ability by maintaining three-dimensional conformation (118). SPR studies have shown that the binding strength of polysaccharides mainly depends on the sulfation degree, but different combinations of glycosidic bonds with different sulfation patterns (such as peroxy-sulfation, nitrogen sulfation, and primary hydroxyl sulfation) may lead to changes in binding strength, thereby affecting the anti-inflammatory response. But there is currently a lack of quantitative affinity data (such as equilibrium dissociation constant Kd values) for specific binding of glycosidic bonds (119, 120). Also, the complexity and heterogeneity of polysaccharide structures (such as variations in monosaccharide composition, glycosidic bond types, and molecular weight) pose significant obstacles to systematic studies of Structure-Activity Relationships (SAR), making it difficult to obtain polysaccharide samples with clear structures. This causes small changes in parameters such as monosaccharide composition and glycosidic bond types to potentially trigger abnormal SAR. Altering the degree of sulfation or molecular weight alone cannot stably regulate activity, and leads to significant activity differences among different batches or variants (121–124). Even in cases where SPR confirms that the degree of sulfation dominates affinity, the large number of polysaccharide components and structural complexity may mask the correlation of glycosidic bonds or conformations, failing to show consistency in specific experiments (125). Therefore, quantifying SAR requires integrating multi-parameter analyses such as sulfation patterns, glycosidic bonds, and conformations, and using multivariate statistical methods such as principal component analysis to analyze and interpret these complex interactions (126, 127).

### 3.2 Comparative analysis of different polysaccharide sources and their relative efficacies

Polysaccharides from various sources such as plants, animals, and microorganisms differ in structure, activity, and application, which directly affect the relative strength of their anti-inflammatory effects. The efficacy comparison is mainly based on chemical composition, structural characteristics (such as molecular weight, monosaccharide residues), and biological activity performance. Among them, plant-derived polysaccharides are the most widely studied category, covering bamboo shoots, longans, and different plant species. Their efficacy is highly dependent on their structure and source part. From the perspective of structure-activity relationship, bamboo shoot polysaccharides have various activities such as anti-diabetic, antioxidant, anti-inflammatory, and immunomodulatory effects due to their water-soluble high molecular weight and monosaccharide composition (128). Longan polysaccharides (especially purified acidic polysaccharides) strongly inhibit inflammation through antioxidant mechanisms, and their activity is affected by the degree of purification; the anti-inflammatory abilities of crude polysaccharides and pure polysaccharides are different (129). In terms of source part, polysaccharides from different plant stems and barks have significant differences in chemical composition, morphological structure, and antioxidant/anti-inflammatory activities. The anti-inflammatory activity of polysaccharides from different parts of the same plant may also change due to the extraction part (such as stem *vs*. bark), indicating that the source part is a key determinant of their relative efficacy (130).

Overall, plant polysaccharides have diverse biological activities (such as immune enhancement, antioxidant), and structural characteristics such as molecular weight, glycosidic bond type, and degree of branching directly affect their activity (131). They also have the advantages of wide availability, safety, and ease of modification, and have strong anti-inflammatory effects in intestinal and chronic inflammation. But there are large differences in activity among different species (such as Longan *vs*. *Cistanche*), and structural complexity may also limit the standardization of some applications (132, 133). Relatively speaking, animal-derived polysaccharides have been less studied but have unique efficacy. For example, polysaccharides extracted from maggots (MEs) are composed of glucose, mannose, etc., and have potential anti-colon cancer effects and related anti-inflammatory effects, but their molecular mechanism is unclear (134), suggesting that they may have advantages in specific cancer-related inflammation. But compared with plant polysaccharides, the SAR of animal-derived polysaccharides requires more research and analysis, so their efficacy is weaker and their applications are limited (135). Microbial and fungal-derived polysaccharides include sulfated polysaccharides from *P. cocos* and fungal polysaccharides such as *D. indusiata*, which usually have high efficacy but are structurally sensitive. Sulfated polysaccharides from *P. cocos* have anti-inflammatory and anti-cancer activities, and their efficacy is affected by monosaccharide residue composition (a key factor) and degree of sulfation; different structures lead to differences in anti-inflammatory efficacy (136). *D. indusiata* polysaccharides have anti-inflammatory, immunomodulatory, and antioxidant effects, with strong effects in *in vitro* and *in vivo* models, and their structural complexity gives them great potential for nano-applications (137). In terms of relative efficacy, microbial polysaccharides often have enhanced biological activity due to modifications (such as sulfation), and their anti-inflammatory effects may be better than plant polysaccharides (e.g., more precise in targeting inflammatory signaling pathways). But limited by their sources and complex extraction processes, they are slightly inferior to plant-derived polysaccharides in terms of accessibility (136).

### 3.3 Key anti-inflammatory mechanisms

#### 3.3.1 Regulation of immune cell function

Natural polysaccharides exert anti-inflammatory effects by regulating immune cell functions, with structural-function relationships reflected in specific examples. Astragalus polysaccharides (APS) reverse the decreasing trend of CD107a (a marker indicating the inhibition of CD8^+^ T cell activation) in inflammatory colorectal cancer models, inhibit the expression of STAT3 and activated Gal-3, and further reduce the expression of LAG3 in tumor-infiltrating CD8^+^ T cells—enhancing the killing ability of CD8^+^ T cells. In Ulcerative Colitis (UC) models, APS also restores the balance of Th17/Treg cells (decreasing Th17 cells and increasing Treg cells) by inhibiting the abnormal activation of the TIGIT/CD155 signaling pathway and reducing the protein levels of PI3K, AKT, and p-AKT (138–140). Currently, most mechanistic studies remain at the level of pathway protein expression, with no clarification of direct binding sites between polysaccharides and target proteins, and insufficient analysis of cross-talk between multiple pathways. *Tetrastigma hemsleyanum* polysaccharides (THP) reduce the body temperature of dry yeast-induced febrile mice, lower the levels of Prostaglandin E2 (PGE2) and cAMP, and decrease the levels of thyroid hormones such as Thyrotropin-Releasing Hormone (TRH), Thyroid-Stimulating Hormone (TSH), T3, and T4. Histologically, THP effectively reduces the degree of inflammatory cell infiltration in liver and hypothalamus tissues and alleviates tissue damage, which is achieved by inhibiting the TLR4/MyD88/NF-κB signaling pathway (decreasing the mRNA and protein levels of TLR4, MyD88, IKKα, IKKβ, IKKγ, IκB-α, NF-κB, and NF-κB p65) (141–145).

#### 3.3.2 Balanced regulation of oxidative stress and inflammation

*Selaginella uncinata* polysaccharide SUSP-4 significantly improves the histological appearance of the colon in Inflammatory Bowel Disease (IBD) models, upregulates the expression of key intestinal barrier proteins (Occludin and ZO-1), and suppresses macrophage activation (downregulating the expression levels of CD68 and CD86). It also reduces the serum levels of Myeloperoxidase (MPO) and Malondialdehyde (MDA), upregulates the levels of Catalase (CAT) and Total Superoxide Dismutase (T-SOD), and increases the expression of Nrf2 while decreasing the expression of Cyclooxygenase-2 (COX-2) and p-NF-κB—alleviating oxidative stress by breaking the Keap1-mediated inhibitory regulation of Nrf2 (146–149). Alhagi honey polysaccharide AHPN80 effectively promotes the activity of Alcohol Dehydrogenase (ADH) and Aldehyde Dehydrogenase (ALDH), inhibits the activity of Alanine Aminotransferase (ALT) and Aspartate Aminotransferase (AST), increases High-Density Lipoprotein (HDL) levels, and reduces Low-Density Lipoprotein (LDL), Total Cholesterol (TC), and Triglyceride (TG) levels to alleviate liver damage in Alcoholic Liver Disease (ALD) models. It also reverses the reduction in SOD activity, decreases LPS levels, reduces the accumulation of toxic metabolites in the liver, and inhibits the TLR4/MAPK pathway (lowering the protein expression levels of TLR4, MyD88, p-p38, p-ERK, and p-JNK) to reduce the levels of pro-inflammatory factors (TNF-α, IL-6, IL-1β) and ROS (150–155).

#### 3.3.3 Mucosal barrier repair and gut microbiota regulation

*Morus alba* polysaccharide (Mup) relieves pain in Knee Osteoarthritis (KOA) models (confirmed by gait analysis and visual assessment of knee joint swelling), restores damaged trabecular bone morphology, and ameliorates the KOA-induced disordered arrangement of chondrocytes by inhibiting the upregulation of MMP-3 and MMP-13. It also reduces the levels of pro-inflammatory cytokines (TNF-α, IL-6, NO) and restores KOA-induced gut microbiota dysbiosis (with the most significant effect at a dose of 200 mg/kg) (156–160). Pectic polysaccharides alter specific gut microbiota, repair the intestinal mucosal barrier, and reshape the microbial metabolome. Microbial metabolites act on receptors (e.g., TLRs, Epidermal Growth Factor Receptor (EGFR), GPR43) on the surface of immune cells or tissue cells, activating multiple signaling pathways to regulate immune responses and tissue function, thereby alleviating inflammatory symptoms, balancing pro-inflammatory/anti-inflammatory mediators, and repairing tissues (161).

#### 3.3.4 Synergistic anti-inflammation with drugs/probiotics

**Figure 1:**
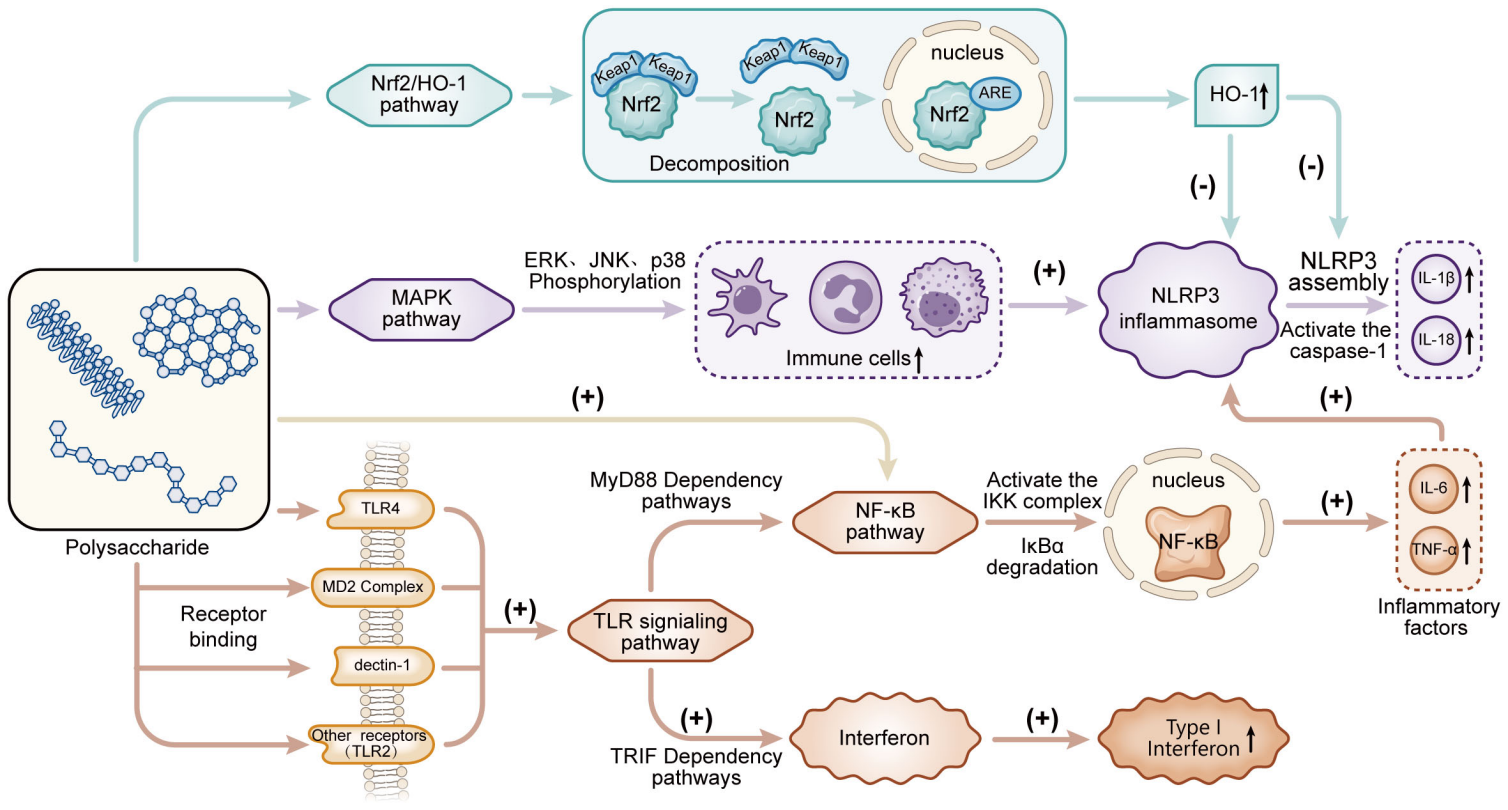
The regulatory mechanisms of polysaccharides on inflammatory signaling pathways and immune modulation. External stimuli such as polysaccharides activate multiple signaling pathways including Nrf2/HO-1, MAPK, and TLR-dependent (MyD88 and TRIF pathways) NF-κB signaling, regulating immune cell proliferation, inflammatory factor secretion, and NLRP3 assembly. These pathways interact to modulate the activation of caspase-1, the release of cytokines (e.g., IL-1β, IL-18, IL-6, TNF-α), and the induction of type I interferon, thereby orchestrating the inflammatory response and immune regulation.

Natural polysaccharides also achieve synergistic anti-inflammation through combination with traditional drugs or probiotics. When combined with NSAIDs, polysaccharides such as Lycium barbarum polysaccharide (LBP) (which inhibits the NF-κB pathway to reduce pro-inflammatory factors) and *Auricularia auricula* polysaccharide (AAP) (which enhances intestinal barrier function by promoting Occludin/ZO-1 expression and regulates flora) complement the COX-2/PGE2 pathway inhibition of NSAIDs, reducing the required dose of single drugs and repairing NSAID-induced mucosal erosion (162). When combined with probiotics (e.g., *Bifidobacterium*, *Lactobacillus*), polysaccharides such as soybean polysaccharides and *Pueraria lobata* polysaccharide (PKP) provide fermentation substrates to promote probiotic colonization and SCFA production (SCFAs enhance intestinal barrier function by activating the GPR43 receptor and upregulating tight junction proteins). Probiotics also metabolize polysaccharides into low-MW bioactive fragments to improve bioavailability, and together they activate Treg cells to secrete IL-10 and inhibit the TLR4/MyD88 pathway, forming a closed-loop regulation of “polysaccharides → flora proliferation → increased SCFAs → immune homeostasis → inflammation alleviation” (162). Natural polysaccharides exert anti-inflammatory effects through multiple mechanisms, including regulating immune cell function, balancing oxidative stress and inflammation, repairing the mucosal barrier, modulating gut microbiota, and synergizing with drugs/probiotics (**Figure 1**). This reflects their advantage of multi-targeted and multi-pathway synergistic anti-inflammation, providing a safe and diverse natural strategy for the prevention and treatment of inflammatory diseases; however, the direct binding sites between polysaccharides and target proteins, as well as the crosstalk mechanisms among multiple pathways, remain unclear. Future breakthroughs can be made in analyzing molecular interaction mechanisms, modifying nano-delivery systems, and conducting large-sample clinical evidence-based studies to further promote their transformation into personalized anti-inflammatory therapeutic agents.

## 4 Nanonization strategies: efficacy enhancement

### 4.1 Nanonization-mediated property optimization

Ordinary natural polysaccharides face challenges of low bioavailability due to poor water solubility and susceptibility to digestive enzyme degradation, and nanonization overcomes these limitations through multiple mechanisms. Nanonization increases the specific surface area of polysaccharides and exposes hydrophilic groups to enhance aqueous dispersibility, while reducing digestive enzyme degradation via steric hindrance. For example, polysaccharide nanocarriers improve the gastrointestinal stability, processing performance, and digestive tolerance of active ingredients (163–165), and enable controlled release to prolong intestinal retention and improve delivery efficiency to target organs (166). The nanoscale particle size (usually <200 nm) enables passive targeting via the EPR effect or M cell transport (e.g., chitooligosaccharide nanospheres are absorbed by M cells to improve oral bioavailability and accumulate in the liver (167)). Active targeting can be achieved through ligand modification or pH-responsive design (e.g., modification enhances the targeting of Lycium barbarum polysaccharides to inflammatory macrophages, improving their liver targeting and anti-inflammatory effects (168, 169)) and can also prevent gastric acid degradation and promote intestinal epithelial uptake (170). Nanonization also regulates pharmacokinetics to enhance efficacy and reduce toxicity: Polyethylene Glycol (PEG) modification avoids recognition by the mononuclear phagocyte system (MPS) to prolong the blood drug half-life (168), and nanocarriers enable sustained release to maintain effective concentrations (e.g., polysaccharide nanosystems loaded with flavonoids improve the oral bioavailability of hesperidin (171–173), and chitosan nanoparticles increase bioavailability and alleviate liver fibrosis damage (167)). Also, nanonization overcomes structural limitations, such as regulating MW to avoid gastrointestinal degradation and aiding in the analysis of pharmacodynamic mechanisms (e.g., revealing the metabolic effect of gut microbiota on active substances with low bioavailability (174)).

### 4.2 Anti-inflammatory mechanisms of nanopolysaccharides

#### 4.2.1 Precise regulation of immune cells

Nanopolysaccharides achieve precise regulation of immune cells to alleviate inflammation (**Table 2**). For macrophages—core cells in inflammation and immune regulation—nanopolysaccharides can directly reprogram their functions as immune adjuvants: polysaccharide nano-adjuvants target receptors on the macrophage surface, inducing polarization from M1 to M2, reducing the production of pro-inflammatory factors such as TNF-α and IL-6, and increasing the secretion of anti-inflammatory factors such as IL-10 to improve immunotherapeutic efficacy (194). Chitosan nanoparticles (CS NPs) of different MWs can activate the Stimulator of Interferon Genes (STING)-mediated autophagy or NLRP3 signaling pathway, enhancing macrophage immune responses and reducing the release of pro-inflammatory factors (195). They can also synergistically inhibit pro-inflammatory activity through multiple pathways such as cGAS-STING, TLRs, and cell death signaling to alleviate inflammation and tissue damage (196). For neutrophils—main recruited cells in the early stage of inflammation—nanopolysaccharides primarily regulate them through indirect effects: through the design parameters of nanomaterials (size, shape, charge, surface modification), they inherently interact with myeloid cells such as neutrophils, inhibiting their chemotaxis and infiltration and reducing migration to inflammatory sites (197). They can also interfere with the neutrophil-macrophage feedback amplification axis (e.g., blocking signal transduction between the two cell types to reduce neutrophil infiltration in acute inflammation models (198)) or reduce the pro-inflammatory phenotype and infiltration level of neutrophils by analyzing surface gene regulatory pathways via single-cell RNA sequencing (scRNA-seq) (199).

**Table 2:** Polysaccharide and immune cell interactions anti-inflammatory mechanisms.

Name	Immune cells	Mechanism	Type	References
Aminoglycan layered hydrogel	Macrophages	Recruit and induce M2 polarization, promote angiogenesis and cardiomyocyte survival, and alleviate inflammation after Myocardial Infarction (MI)	*In vivo*	(175)
Fucoidan (*Fucus vesiculosus*)	Monocytes/Hepatic macrophages	Bind to Prolyl Hydroxylase Domain Protein 2 (PHD2), promote Hypoxia-Inducible Factor-1α (HIF-1α) hydroxylation and degradation, inhibit infiltration of inflammatory monocytes, and improve Metabolic-Associated Alcoholic Liver Disease (MetALD)	*In vivo*	(176)
*Tetrastigma hemsleyanum* polysaccharide (THP)	Macrophages	Inhibit NLRP3-Caspase-1-Gasdermin D (GSDMD) signaling, block macrophage pyroptosis and IL-1β secretion, and alleviate Acute Lung Injury (ALI)	*In vivo*	(177)
Injectable self-healing Fucoidan-Hydrazone hydrogel	Macrophages	Retain Fucoidan to drive M2 polarization, with antioxidant, anti-inflammatory, and good tissue compatibility	*In vivo*	(178)
*Codonopsis pilosula* acidic polysaccharide (CPAP)	T cells/Neutrophils	Enrich gut flora *Lactobacillus* → activate T cell anti-tumor immunity and inhibit neutrophil degranulation inflammation	*In vivo*	(179)
Gellan gum–tannic acid three-network polysaccharide hydrogel	Macrophages	Triple cross-linking for antibacterial, anti-oxidation, and hemostatic properties; regulate inflammation via M2 polarization and repair diabetic infected wounds	*In vivo*	(180)
*Atractylodes macrocephala* polysaccharide (PAMK)	Macrophages	Regulate long non-coding RNA (lncRNA) GAS5/microRNA (miR)-223-3p/NLRP3 axis to reduce macrophage pyroptosis and alleviate LPS-induced inflammation	*In vivo*	(181)
DEPS (*Cinnamomum burmannii* endophytic fungal polysaccharide)	Macrophages/Neutrophils	Downregulate IL-6, inhibit neutrophil migration, upregulate IL-10, and protect against Acetaminophen (APAP)-induced liver injury	*In vivo*	(182)
Sulfated saikosaponin DPI + Dihydromyricetin (DHM) composite nanoparticles	Inflammatory monocytes	Target macrophages to scavenge ROS, deliver Naringenin to inhibit inflammatory signals, and enhance cytoprotective effects	*In vivo*	(183)
Fucoidan (review, UC)	Macrophages/T cells	Inhibit NF-κB/MAPK, enhance tight junction proteins, and reshape gut microbiota-SCFA axis	*In vivo*	(184)
*Panax notoginseng* polysaccharide microspheres (FA-PPi-Ms)	Macrophages	Target JAK2-STAT3 inhibition, promote M2 polarization, and delay the progression of Rheumatoid Arthritis (RA)	*In vivo*	(185)
Fermented *Polygonatum* polysaccharide (FPKP_3_)	RAW264.7 macrophages	Improve structural conversion and Peroxisome Proliferator-Activated Receptor γ (PPARγ) binding affinity, inhibit inflammation and promote M1→M2 polarization, and ameliorate obesity-related inflammation	*In vitro*	(186)
*Paeonia alba* polysaccharide-iron nanocapsules (PPFeCs)	Macrophages	Regulate metabolism via PI3K/Akt, promote Oxidative Phosphorylation (OXPHOS) and inhibit NF-κB, drive M2 polarization, and treat IBD-Iron Deficiency Anemia (IDA)	*In vivo*	(187)
Edible fungal polysaccharides (systematic review)	Macrophages/Dendritic cells/T-B cells	Structure-function coupling: synergistic immune regulation via multiple pathways (TLR4/MyD88/NF-κB, NLRP3, MAPK, etc.)	*In vivo*	(188)
*Solanum tuberosum* L. polysaccharide (STP)	THP-1 macrophage-like cells	Downregulate IL-1β/IL-6/TNF, activate NRF2 and BCL2/BAX, and exhibit multi-target anti-inflammatory effects	*In vitro*	(189)
*Spirulina subsalsa* polysaccharide hydrogel (SPS)	Macrophages	Promote NO production, inhibit TNF-α and increase IL-10; self-assembled hydrogel with excellent immunotherapeutic potential	*In vivo*	(190)
*Portulaca oleracea* acidic heteropolysaccharide (POPAA-1)	Alveolar macrophages	Block LPS-TLR4 binding, inhibit NF-κB and NLRP3-Caspase-1-GSDMD pyroptosis pathway, and improve sepsis-induced lung injury	*In vivo*	(191)
ROS-responsive polysaccharide injectable hydrogel	Macrophages	Dynamic boronic acid ester network scavenges ROS, promotes M2 polarization and antibacterial activity, and facilitates breast bone repair	*In vivo*	(192)
*Antrodia cinnamomea* highly sulfated α-1,4-Galactoglucan	Macrophages	Downregulate phosphorylation of AKT/ERK/EGFR/Focal Adhesion Kinase (FAK) and TGFβRII expression, and significantly inhibit LPS-induced inflammation	*In vivo*	(193)

**Figure 2:**
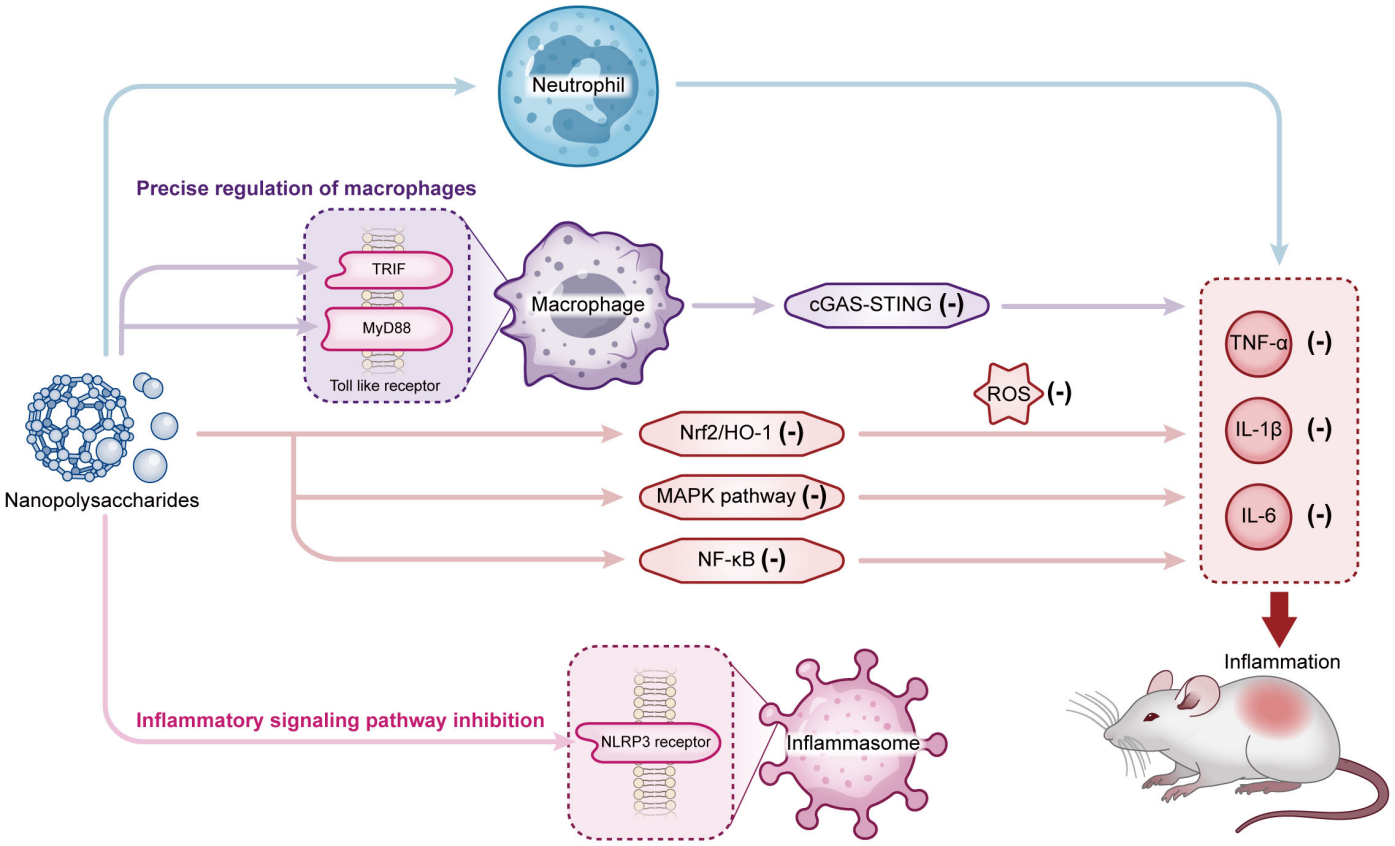
The regulatory mechanisms of nanopolysaccharides on inflammatory response via multi-pathway inhibition and immune cell modulation. External stimuli such as nanopolysaccharides exert anti-inflammatory effects by precisely regulating macrophages (via toll-like receptor signaling pathways MyD88 and TRIF), inhibiting multiple inflammatory signaling pathways (NF-κB, MAPK, Nrf2/HO-1, cGAS-STING) and NLRP3, thereby reducing the release of pro-inflammatory cytokines (TNF-α, IL-1β, IL-6) and regulating immune cells like neutrophils. These coordinated actions collectively suppress the inflammatory response.

Nanopolysaccharides exert anti-inflammatory effects by inhibiting NF-κB, MAPK inflammatory pathways to reduce pro-inflammatory factor release, activating Nrf2/HO-1 antioxidant pathway to alleviate oxidative stress, and regulating NLRP3, interfering with TLR signal axis and other multiple mechanisms synergistically (**Figure 2**). They combine the safety of natural polysaccharides with the targeting and sustained-release properties of nanotechnology, showing significant advantages in the synergy of anti-inflammatory mechanisms and clinical translation. Although the anti-inflammatory differences among different modifications and molecular weights, as well as the details of pathway interactions, still need in-depth exploration, they provide a highly promising direction for the innovative development of natural-derived anti-inflammatory agents and personalized treatment of inflammatory diseases.

#### 4.2.2 Targeted blockade of inflammatory signaling pathways

Nanopolysaccharides block the activation of inflammatory signaling pathways to suppress the inflammatory cascade (**Table 3**). In inhibiting the NF-κB pathway, CS NPs can inhibit IκB-α degradation, block NF-κB activation, and reduce the expression of pro-inflammatory factors such as TNF-α and IL-1β (212). APS nanoparticles (AU) can significantly reduce the protein level of phosphorylated NF-κB (p-NF-κB) relative to total NF-κB, decreasing the release of downstream inflammatory mediators (213). Nanoscale *Ganoderma lucidum* polysaccharides (GLPS) further block NF-κB activation by inhibiting TLR4-mediated MyD88-TRAF6 signaling (214). Nano-chitosan can also indirectly inhibit NF-κB signal transduction by activating the STING pathway (212). In regulating the MAPK pathway, nanoscale analogs of tremella polysaccharides (e.g., GLPS) can significantly downregulate the phosphorylation levels of ERK, JNK, and p38 MAPK. Experiments confirm that GLPS can reduce p38 phosphorylation by more than 60%, blocking the MAPK/NF-κB signal axis (214). CS NPs can also reduce the release of pro-inflammatory mediators by inhibiting p38 MAPK phosphorylation (IC_50_=1.95 μM) (215). Some nanopolysaccharides (e.g., PF543) can simultaneously inhibit the activation of p38 MAPK and NF-κB p65, confirming the synergistic regulation between the two pathways (216, 217). In the ethanol-induced gastric ulcer model, nanoparticles can block the inflammatory cascade by inhibiting the p38 MAPK/NF-κB/NLRP3 signal axis (218). In activating the Nrf2/HO-1 antioxidant pathway, AU can activate the nuclear factor Nrf2, promote the expression of downstream HO-1, and scavenge ROS in the inflammatory area to alleviate oxidative stress damage (214). In the Diethyl Phthalate (DEHP)-induced inflammation model, nanopolysaccharides can also inhibit MAPK/NF-κB activation through the Nrf2/HO-1 pathway, blocking pyroptosis (217). Chitosan nanocarriers can simultaneously regulate the NF-κB/Nrf2/HO-1/MAPK pathway, forming a multi-pathway synergistic anti-inflammatory network (219–221). Also, nano-chitosan can block NLRP3 activation and IL-1β maturation by inhibiting the NADPH oxidase/MAPK/NF-κB axis (222). Sulfated modified nanopolysaccharides (e.g., SH-modified) can inhibit the NF-κB/MAPK pathway through the TLR-MyD88-TRAF6 axis to enhance immune regulation (223). High-MW CS NPs can enhance adjuvant activity by activating STING-mediated autophagy and NLRP3 signaling (213), and polysaccharide nanocarriers can improve drug bioavailability and extend anti-inflammatory effects through sustained release (224).

**Table 3:** Anti-inflammatory signaling pathways of polysaccharides.

Name	Signaling pathways	Mechanism	References
β-glucan	Dectin-1 → Syk → NOX-2/ROS-Lipidated LC3 (LAP); NF-κB	Trigger LC3-related phagocytosis, clear inflammatory debris and inhibit NF-κB, improve chronic inflammation	(200)
β-glucan	Interferon (IFN)-I/JAK-STAT	“Trained immunity 2.0” —— Reprogram hematopoietic stem cells, make descendant granulocytes M2-like anti-inflammatory phenotype	(201)
Chitosan (COS, Degree of Polymerization (DP) = 6)	TLR2 → NF-κB	Block TLR2 dimerization, inhibit iNOS/IL-6/TNF-α production	(202)
Chitosan nanoparticles	cGAS-STING-Interferon Regulatory Factor 3 (IRF3); NF-κB	Transiently activate STING to promote antigen presentation, then Suppressor of Cytokine Signaling 1 (SOCS1) negatively feedback inhibits NF-κB	(203)
Chitosan	mammalian Target Of Rapamycin (mTOR)-HIF-1α	Inhibit microglial Warburg metabolism, alleviate neural inflammation	(204)
Alginate	TLR4-MyD88-NF-κB; p38/JNK	Scavenge ROS, inhibit NF-κB & MAPK, reduce IL-1β/IL-6	(205)
Double-layer alginate hydrogel	JAK1/STAT3; Vascular Endothelial Growth Factor (VEGF)-signaling	First release IL-10 to reduce inflammation, then release VEGF to promote neovascularization and accelerate diabetic wound healing	(206)
Alginomannan (AOS)	NF-κB; JNK; Gut microbiota-SCFA	Regulate flora → increase butyrate, synergistically inhibit NF-κB/JNK, protect colitis	(207)
Fucoidan (SCVP-2)	NF-κB; ERK	Reduce hepatic IL-1β/TNF-α, improve metabolic inflammation	(208)
Fucoidan	Takeda G Protein-Coupled Receptor 5 (TGR5)-cAMP-Protein Kinase A (PKA)	Induce macrophage M2 polarization, alleviate obesity-related inflammation	(209)
Fucoidan	Nrf2/HO-1	Elevate antioxidant enzymes, reduce chronic nephritis oxidative stress	(210)
Pectin	NLRP3; NF-κB; MAPK	Inhibit inflammasome assembly, reduce iNOS/COX-2, restore intestinal barrier	(211)

#### 4.2.3 Protection of mucosal barrier in inflammatory tissues

Nanopolysaccharides protect the mucosal barrier of inflammatory tissues through physical and chemical mechanisms. In physical barrier construction, polysaccharides such as nano-chitosan can form a dense “nanomembrane” on the mucosal surface through electrostatic adsorption or hydrophobic interactions. This barrier effectively blocks the contact between pathogenic bacteria (e.g., *E. coli*), endotoxins (LPS), and epithelial cells, reducing the activation of inflammatory signaling pathways such as TLR4/NF-κB and thereby decreasing the release of pro-inflammatory factors such as TNF-α and IL-1β (225–227). In intestinal inflammation models, such nanomembranes can significantly reduce mucosal damage area (228). Polysaccharide nanoparticles such as AMP can promote goblet cells to secrete mucin MUC2, increasing mucus layer thickness and integrity to repair damaged mucus barriers (228). nanostructures can also indirectly maintain mucus layer homeostasis by regulating gut microbiota (e.g., increasing the abundance of anti-inflammatory bacteria such as *Muribaculaceae* (229)).

In chemical repair and regeneration, hyaluronic acid nanoparticles can activate CD44 receptors or integrin signaling, accelerating epithelial cell migration and wound closure (230, 231). Polysaccharides such as Huangshui polysaccharide (NLS-2) can upregulate the expression of tight junction proteins such as Occludin, Claudin-1, and ZO-1 to repair the intestinal epithelial mechanical barrier (232–235). They can also regulate inflammation by inhibiting the TLR4/NF-κB pathway (e.g., lactic acid promotes macrophage M2 polarization to block the “inflammation-barrier damage” vicious cycle (236, 237)) and regulating the STAT3 pathway (e.g., 2’-fucosyllactose (2’-FL) inhibits STAT3 phosphorylation to alleviate colitis (238)). Meanwhile, polysaccharide nanoparticles such as Huangshui polysaccharide and SMSP2 can scavenge ROS to inhibit the destruction of tight junctions by oxidative stress (239) and balance Th1/Th2 responses by regulating macrophage polarization and Treg cell activation to alleviate mucosal immune barrier damage (240). This barrier protection exhibits cross-organ applicability: in the intestinal tract, nanopolysaccharides exert protective effects on UC and sepsis-induced intestinal injury by repairing intestinal epithelial tight junctions, restoring mucus barrier function, and regulating flora homeostasis (241, 242). In the respiratory tract, chitosan nanoparticles can penetrate the tight junctions of respiratory epithelium, enhancing mucosal vaccine delivery efficiency and inducing local immune responses (e.g., promoting secretory Immunoglobulin A (sIgA) secretion) to block pathogen invasion (243).

#### 4.2.4 Balanced regulation of gut microbiota

Nanopolysaccharides regulate gut microbiota balance to enhance anti-inflammatory effects. As “prebiotics,” they can selectively regulate flora composition, increasing the abundance of beneficial bacteria such as *Bifidobacterium* and *Lactobacillus* while inhibiting the overgrowth of harmful bacteria such as *Proteobacteria* and *Fusobacteria*. High-MW nanopolysaccharides exhibit better flora-regulating effects than low-MW ones due to their stronger colonic fermentation capacity (244–247). After fermentation by gut microbiota, nanopolysaccharides significantly increase the production of SCFAs such as butyrate and propionate. SCFAs exert anti-inflammatory effects and maintain intestinal barrier integrity, while also reducing the levels of harmful metabolites such as endotoxins (e.g., LPS) (inulin-based nanopolysaccharides reduce endotoxins by 40% in animal models (248–250)) and alleviating enteritis induced by pro-inflammatory bile acids such as deoxycholic acid (DCA) through the gut microbiota-farnesoid X receptor (FXR) signaling pathway (251, 252). This flora regulation further promotes barrier repair: nanopolysaccharides enhance the physical barrier by increasing tight junction protein expression and repair the immune barrier by reducing the secretion of pro-inflammatory factors such as TNF-α and IL-6, while directly alleviating mucosal oxidative stress and inflammatory responses by inhibiting the NF-κB pathway and reducing ROS levels (253–256). Also, the nano-scale size endows polysaccharides with higher bioavailability, enabling targeted delivery to inflamed intestinal sites for precise regulation of local flora and immune microenvironments (257–259).

### 4.3 Efficacy verification via animal models

Animal models are primarily used to induce inflammatory states for verifying the efficacy of nanopolysaccharides, with common types including chemically induced, infection/toxin-induced, immunosuppressive, and transgenic/condition-specific models. Chemically induced models are widely applied: the Dextran Sulfate Sodium (DSS)-induced colitis model simulates human UC, reproducing intestinal barrier destruction, inflammatory cell infiltration, and flora dysbiosis—researchers evaluate the symptom-alleviating effects of nanocomposites such as polysaccharide-based nanoparticles by monitoring changes in body weight, colon length, and inflammatory markers (260–263). The High-Fat Diet (HFD)-induced obesity and inflammation model is used to investigate the effects of polysaccharide fractions on flora composition (264). Infection or toxin-induced models use LPS in mouse models to verify the protective effect of Galacto-Oligosaccharides (GOS) (265); the *Listeria monocytogenes* infection model tests the anti-infective capacity of bifidocin A, including its effects on barrier function and inflammatory responses (266); the rotenone-induced model explores the link between intestinal inflammation and neurological disorders such as movement disorders (267).

Immunosuppressive models involve Cyclophosphamide (CTX)-induced intestinal mucosal damage and immunosuppression in mice (simulating chemotherapy or immunodeficiency states), which are used to evaluate the effects of polysaccharide formulations on flora diversity and oxidative stress (268, 269). Transgenic or condition-specific models include germ-free mouse models (verifying the necessity of gut microbiota in inflammatory mechanisms—e.g., confirming the dependence of TNF-driven inflammation on flora in RA research (270)); “pseudo-germ-free” mice (with flora depleted via antibiotics) (assessing whether the inflammatory regulation of quinic acid depends on flora (271)); and chicken intestinal inflammation models (studying the regulation of Th17/Treg balance by flora via Fecal Microbiota Transplantation (FMT) (272, 273)).

## 5 Clinical translation potential and challenges

### 5.1 Clinical prospects

#### 5.1.1 Clinical application prospects in major inflammation-related diseases

Natural polysaccharides (especially nanopolysaccharides) show broad clinical potential in chronic inflammation-related diseases. In intestinal inflammatory diseases (UC, Crohn’s disease), nanopolysaccharides repair the intestinal barrier, regulate gut microbiota, and inhibit the NF-κB/MAPK pathways—DSS-induced colitis models confirm their ability to reduce mucosal damage and inflammatory factor levels, laying the foundation for UC treatment (267, 268). In joint diseases (RA, KOA), modified *Panax notoginseng* polysaccharide microspheres target JAK2-STAT3 to inhibit pro-inflammatory signals and promote macrophage M2 polarization (260), while Mup restores KOA cartilage and subchondral bone structure by inhibiting MMP-3/MMP-13 and regulating flora —providing new options for arthritis treatment. In liver diseases (ALD, non-alcoholic fatty liver disease), AHPN80 inhibits the TLR4/MAPK pathway to reduce liver inflammation and oxidative stress (154), while fucoidan reduces hepatic IL-1β/TNF-α levels to improve metabolic inflammation (257)—showing promise for liver inflammatory disease intervention. In respiratory diseases (ALI, asthma), nanoscale GLPS inhibits the PI3K/Akt/NF-κB pathway to alleviate ALI (214), while chitosan nanoparticles penetrate respiratory epithelial tight junctions to enhance mucosal immunity (e.g., promoting sIgA secretion) and block pathogen invasion (242, 243)—offering new strategies for respiratory inflammation. Also, the “polysaccharide-traditional anti-inflammatory drug/probiotic” synergistic system reduces NSAID-induced mucosal damage (261–265) and enhances probiotic stability and colonization (266, 267), providing a basis for combined clinical therapies.

#### 5.1.2 Current status of clinical trials on polysaccharide drugs

The clinical translation of polysaccharides and their nano-formulations in the treatment of inflammatory diseases faces multiple challenges: Firstly, their low bioavailability severely limits their application in fields such as Cardiovascular Diseases (CVD), and there is an urgent need to improve delivery efficiency through technologies like nano-carriers (268). Secondly, although they show potential in IBD in terms of anti-inflammation, mucosal repair, and microbiota regulation—for example, natural polysaccharides combined with FMT can correct dysbiosis (269), or targeted delivery via nanoparticles can enhance local drug concentration (270)—large-scale clinical trial evidence supporting their efficacy is still lacking (271).

Also, the dual nature of polysaccharides in immunoregulation (e.g., lipopolysaccharides can both induce inflammation and act as vaccine adjuvants) further complicates trial design, requiring precise control of dosage and delivery methods (272). Among the clinical studies that have been advanced, the CVD field is limited by bioavailability and delivery efficiency, and the translation of related nano-carriers is still in the early stages (268); preliminary clinical trials on Chronic Kidney Disease (CKD) have verified its safety (273); while the combination therapy and nano-targeting strategies for IBD are promising but have not yet achieved large-scale clinical validation (271). Nanotechnology provides a direction to break through the above bottlenecks: nano-polysaccharide carriers can improve solubility, prolong release, and reduce systemic toxicity by encapsulating anti-inflammatory drugs (260, 261), while partially overcoming the low bioavailability defect of natural polysaccharides by enhancing cell uptake efficiency (262, 263). For example, in RA, nano-polysaccharides have demonstrated excellent pharmacokinetic properties (264), but their long-term safety and effectiveness in different inflammatory diseases still need further verification through more clinical trials (265). Although animal models are widely used to evaluate the anti-inflammatory activity of polysaccharide nanomedicines, they have significant limitations in simulating the complex physiological environment of humans. Especially in IBD research, animal models cannot fully replicate the dynamic mucosal barrier, microbial interaction network, and immunometabolic differences in the human intestine. This leads to inaccurate predictions of the targeted delivery effect and metabolic kinetics of polysaccharides (266, 267). For example, although polysaccharide nanocarriers (such as chitosan-modified PLGA nanoparticles CS-PIPP) have shown efficacy in DSS-induced mouse colitis models—by regulating gut microbiota (e.g., increasing *Lactobacillus*) and inhibiting inflammatory factors—the heterogeneity of the physicochemical environment and the diversity of flora in the human gastrointestinal tract may weaken their delivery stability (268). In addition, animal models have limited ability to evaluate the duality of immunoregulation (e.g., polysaccharides can both inhibit inflammation and potentially trigger abnormal reactions) and long-term safety (269).

### 5.2 Challenges and future directions

#### 5.2.1 Existing core challenges

Existing studies have shown that polysaccharide nanocarriers can improve bioavailability by 1.5–2.5 times through controlled release and targeted delivery (270, 271), but their long-term physicochemical stability remains unsubstantiated by accelerated experiments and actual storage data. Scale-up production faces challenges such as batch-to-batch variations (272, 273), equipment compatibility, and continuous process development. Additionally, the nanonization of polysaccharide-based materials is sensitive to temperature and shear force, increasing the difficulty of process control (260). Clinical translation must address regulatory barriers including insufficient long-term biosafety data (261), lack of standardized production specifications (e.g., control of residual organic solvents) (260), and compliance risks arising from inadequate stability verification (262). It is necessary to establish a multi-dimensional comparison table to quantify the bioavailability improvement multiples, production feasibility scores, and stability/regulatory limiting factors of each strategy (e.g., liposomes enhance bioavailability by 2–3 times (263)), thereby filling the argumentative gaps in this chapter.

Although current research on the anti-inflammatory effects of natural polysaccharides has clarified the correlation between their structures (monosaccharide composition, molecular weight, glycosidic bond types) and anti-inflammatory efficacy, as well as nanonization optimization strategies, there are still significant research gaps and application obstacles: At the research level, the analysis of structure-activity relationships is superficial, failing to use technologies such as cryo-electron microscopy and molecular docking to clarify the precise impact of subtle structural differences (e.g., sulfate substitution sites, branch chain lengths) on receptor binding and signal transduction (264, 265). Traditional models such as RAW264.7 cells and DSS-induced colitis cannot replicate the superposition of multiple pathological factors (oxidative stress, metabolic disorders, microbiota imbalance) in human chronic inflammation, nor can they simulate the pathological characteristics of special populations (e.g., the elderly, individuals with liver or kidney dysfunction). Furthermore, the regulatory mechanisms of non-immune cells (e.g., fibroblasts) in the inflammation-fibrosis vicious cycle are overlooked (266, 267).

At the application level, ordinary polysaccharides have unstable oral bioavailability due to poor water solubility and susceptibility to degradation by digestive enzymes, while injectable preparations face issues with administration convenience (268). Although nanopolysaccharides have improved water solubility and targeting, they confront prominent core translation challenges: In terms of long-term toxicity, existing short-term experiments have not evaluated the long-term accumulation risks in organs such as the liver and spleen, nor have they investigated genotoxicity, reproductive toxicity, or excessive disturbances to intestinal microbiota homeostasis (269, 270). In terms of immunogenicity, the risks of activating the complement system, inducing the production of specific antibodies, and overactivating immune cells have not been quantified, blurring the boundary between “immunomodulation” and “immune activation”. In terms of industrial scale-up production, raw materials such as plants and algae are greatly affected by the growth environment; laboratory nanonization processes (e.g., ultrasonic emulsification, electrospinning) are difficult to adapt to industrial scales; and there is a lack of online quality monitoring technologies that meet international standards such as USP, EP, and ICH, resulting in significant batch-to-batch quality differences (271). In terms of regulatory pathways, the FDA classifies nanopolysaccharides as “complex drug formulations” and the EMA as “biosimilars”; global regulatory classifications and approval data requirements are inconsistent, and coupled with patent barriers, this leads to confusion in the declaration path.

#### 5.2.2 Future development directions

Future directions focus on addressing these challenges to promote clinical translation. Deepening structure-mechanism analysis: Use advanced technologies such as cryo-electron microscopy to clarify the impact of subtle structural differences (e.g., degree of sulfate substitution, branching pattern) on polysaccharide-receptor binding and pathway regulation. Establish a precise “structure-mechanism” correspondence to guide targeted polysaccharide modification. Standardizing preparation and quality control: Formulate unified national/international standards for polysaccharide extraction, purification, and quality evaluation (e.g., MW distribution, DS, purity). Ensure batch consistency and experimental reproducibility. Optimizing nanocarrier design: Develop smart responsive nanocarriers (e.g., pH-sensitive, ROS-sensitive, enzyme-sensitive) to improve the targeting of nanopolysaccharides to inflammatory sites and realize controlled drug release. Conduct long-term toxicity studies (e.g., chronic exposure, organ accumulation tests) to provide safety data for clinical use. Expanding clinical research and formulation development: Promote multi-center, large-sample RCTs of polysaccharide-drug/probiotic combinations to verify their efficacy and safety in humans. Develop novel formulations (e.g., oral nanoparticles, injectable hydrogels, mucosal sprays) to enhance targeting and patient compliance. Expanding disease application scope: Use systems biology approaches to explore the effects of polysaccharides in understudied fields such as neuroinflammation (e.g., Alzheimer’s disease-related inflammation) and tumor-associated inflammation. Expand the anti-inflammatory application field of polysaccharides.

## 6 Conclusions

This review systematically organizes the anti-inflammatory research progress of natural polysaccharides into four core sections—fundamental mechanisms, natural polysaccharides activities, nanonization strategies, and clinical translation—without redundant pathway-specific subsections. It clarifies the “structure-anti-inflammatory specificity” association of natural polysaccharides (monosaccharide composition, MW, glycosidic bond type determine pathway selection), establishes nanonization as a key solution to improve polysaccharide bioavailability (via solubility enhancement, targeting optimization, and pathway synergy), and constructs a “polysaccharide-drug/probiotic” synergistic anti-inflammatory paradigm (overcoming single-therapy limitations via target complementarity and flora-immune axis regulation). Additionally, it expands the cellular regulatory dimension of polysaccharides, clarifying their role in regulating non-immune cells (endothelial cells, fibroblasts, intestinal epithelial cells) to improve the inflammatory microenvironment. These insights enrich the theoretical system of natural polysaccharides anti-inflammation and provide new directions for the treatment of chronic inflammation-related diseases.

## References

[1] Zhang H Chen H Hu X Muhammad W Liu C Liu W . Inflammation-modulating polymeric nanoparticles: design strategies, mechanisms, and therapeutic applications. eBioMedicine. (2025) 118:105837. doi: 10.1016/j.ebiom.2025.105837, PMID: 40614330 PMC12271758

[2] Godson C Guiry P Brennan E . Lipoxin mimetics and the resolution of inflammation. Annu Rev Pharmacol Toxicol. (2023) 63:429–48. doi: 10.1146/annurev-pharmtox-051921-085407, PMID: 36662584

[3] Li Z Luo B Chen Y Wang L Liu Y Jia J . Nanomaterial-based encapsulation of biochemicals for targeted sepsis therapy. Materials Today Bio. (2025) 33:102054. doi: 10.1016/j.mtbio.2025.102054, PMID: 40688672 PMC12275963

[4] Du Y Chen Y Li F Mao Z Ding Y Wang W . Genetically engineered cellular nanovesicle as targeted DNase I delivery system for the clearance of neutrophil extracellular traps in acute lung injury. Advanced Sci. (2023) 10:2303053. doi: 10.1002/advs.202303053, PMID: 37759381 PMC10646266

[5] Tilg H Adolph TE Tacke F . Therapeutic modulation of the liver immune microenvironment. Hepatology. (2023) 78:1581–601. doi: 10.1097/hep.0000000000000386, PMID: 37057876

[6] Antony F Kinha D Nowińska A Rouse BT Suryawanshi A . The immunobiology of corneal HSV-1 infection and herpetic stromal keratitis. Clin Microbiol Rev. (2024) 37:e00006–24. doi: 10.1128/cmr.00006-24, PMID: 39078136 PMC11391706

[7] Zheng Z Yang J Zheng W Chu Z Wang W Qian H . Comprehensive management of diabetic ulceration: strategies and perspectives. J Controlled Release. (2025) 385:114058. doi: 10.1016/j.jconrel.2025.114058, PMID: 40701270

[8] Taylor EB Hall JE Mouton AJ . Current anti-inflammatory strategies for treatment of heart failure: From innate to adaptive immunity. Pharmacol Res. (2025) 216:107761. doi: 10.1016/j.phrs.2025.107761, PMID: 40348101 PMC12199133

[9] Andretto V Dusi S Zilio S Repellin M Kryza D Ugel S . Tackling TNF-α in autoinflammatory disorders and autoimmune diseases: From conventional to cutting edge in biologics and RNA-based nanomedicines. Advanced Drug Delivery Rev. (2023) 201:115080. doi: 10.1016/j.addr.2023.115080, PMID: 37660747

[10] Song Y Deng Q Li J Chen R Li D Wang S . Structure-activity relationships and mechanisms of natural polysaccharides in modulating neurological disorders via the microbiota-gut-brain axis. Carbohydr Polymers. (2025) 367:123960. doi: 10.1016/j.carbpol.2025.123960, PMID: 40817517

[11] Fu Y Jiao H Sun J Okoye CO Zhang H Li Y . Structure-activity relationships of bioactive polysaccharides extracted from macroalgae towards biomedical application: A review. Carbohydr Polymers. (2024) 324:121533. doi: 10.1016/j.carbpol.2023.121533, PMID: 37985107

[12] Xu W Li X Zhong Y He J Xie W Kang Y . Structural characterizations and antiaging activities of hydrolyzed low-molecular-weight polysaccharides from Polygoni Multiflori Radix Praeparata. Carbohydr Polymers. (2025) 356:123381. doi: 10.1016/j.carbpol.2025.123381, PMID: 40049961

[13] Fan S Zhang Z Zhong Y Li C Huang X Geng F . Microbiota-related effects of prebiotic fibres in lipopolysaccharide-induced endotoxemic mice: short chain fatty acid production and gut commensal translocation. Food Funct. (2021) 12:7343–57. doi: 10.1039/d1fo00410g, PMID: 34180493

[14] Shen Y Zhao H Wang X Wu S Wang Y Wang C . Unraveling the web of defense: the crucial role of polysaccharides in immunity. Front Immunol. (2024) 15:1406213. doi: 10.3389/fimmu.2024.1406213, PMID: 39524445 PMC11543477

[15] Yuan D Li C Huang Q Fu X Dong H . Current advances in the anti-inflammatory effects and mechanisms of natural polysaccharides. Crit Rev Food Sci Nutr. (2022) 63:5890–910. doi: 10.1080/10408398.2022.2025535, PMID: 35021901

[16] Li H Cheng Y Cui L Yang Z Wang J Zhang Z . Combining gut microbiota modulation and enzymatic-triggered colonic delivery by prebiotic nanoparticles improves mouse colitis therapy. Biomaterials Res. (2024) 28:0062. doi: 10.34133/bmr.0062, PMID: 39140035 PMC11321063

[17] Gaikwad D Sutar R Patil D . Polysaccharide mediated nanodrug delivery: A review. Int J Biol Macromolecules. (2024) 261:129547. doi: 10.1016/j.ijbiomac.2024.129547, PMID: 38278399

[18] Zhang Q Wang Y Zhu J Zou M Zhang Y Wu H . Specialized pro-resolving lipid mediators: a key player in resolving inflammation in autoimmune diseases. Sci Bulletin. (2025) 70:778–94. doi: 10.1016/j.scib.2024.07.049, PMID: 39837719

[19] Wang H Kim SJ Lei Y Wang S Wang H Huang H . Neutrophil extracellular traps in homeostasis and disease. Signal Transduction Targeted Ther. (2024) 9:1933. doi: 10.1038/s41392-024-01933-x, PMID: 39300084 PMC11415080

[20] Victoria Vaglienti M Paz MC Victoria Gutierrez M Virginia Subirada P Luna J Bonacci G . Nitro-Oleic acid protects from neovascularization, oxidative stress, gliosis and neurodegeneration in oxygen-induced retinopathy. Redox Biol. (2025) 83:103634. doi: 10.1016/j.redox.2025.103634, PMID: 40273475 PMC12051658

[21] Ioannidis M Tjepkema J Uitbeijerse MR van den Bogaart G . Immunomodulatory effects of 4-hydroxynonenal. Redox Biol. (2025) 85:103719. doi: 10.1016/j.redox.2025.103719, PMID: 40489926 PMC12174603

[22] Song J Zhang Y Bai Y Sun X Lu Y Guo Y . The deubiquitinase OTUD1 suppresses secretory neutrophil polarization and ameliorates immunopathology of periodontitis. Advanced Sci. (2023) 10:2303207. doi: 10.1002/advs.202303207, PMID: 37639212 PMC10602526

[23] Fredman G Serhan CN . Specialized pro-resolving mediators in vascular inflammation and atherosclerotic cardiovascular disease. Nat Rev Cardiol. (2024) 21:808–23. doi: 10.1038/s41569-023-00984-x, PMID: 38216693 PMC12863063

[24] Chi Y Jiang H Yin Y Zhou X Shao Y Li Y . Macrophage signaling pathways in health and disease: from bench to bedside applications. MedComm. (2025) 6:70256. doi: 10.1002/mco2.70256, PMID: 40529613 PMC12171086

[25] Jiang S Wang D Zou C Zhu Z Luo C Wang Z . Macrophages at the crossroads of cellular senescence and cancer development and progression: Therapeutic opportunities and challenges. Pharmacol Ther. (2025) 274:108906. doi: 10.1016/j.pharmthera.2025.108906, PMID: 40782901

[26] Liang L Xu W Shen A Fu X Cen H Wang S . Inhibition of YAP1 activity ameliorates acute lung injury through promotion of M2 macrophage polarization. MedComm. (2023) 4:293. doi: 10.1002/mco2.293, PMID: 37287755 PMC10242261

[27] Hu Y Liu J Xu M Pu K . Dual-locked fluorescence probe for monitoring the dynamic transition of pulmonary macrophages. J Am Chem Society. (2025) 147:7148–57. doi: 10.1021/jacs.5c00506, PMID: 39946549

[28] Liu H Wang H Li Q Wang Y He Y Li X . LPS adsorption and inflammation alleviation by polymyxin B-modified liposomes for atherosclerosis treatment. Acta Pharm Sin B. (2023) 13:3817–33. doi: 10.1016/j.apsb.2023.06.005, PMID: 37719368 PMC10501887

[29] Zhang Q Zhang H Feng S Yao M Ding J Li X . Macrophage metabolic reprogramming ameliorates diabetes-induced microvascular dysfunction. (2024). doi: 10.2139/ssrn.4960120, PMID: 39647239 PMC11667058

[30] Li D Yang T Li Y Lyu X Hu C Yan J . Adipose tissue macrophages as initiators of exacerbated periodontitis in estrogen-deficient environments via the amplifier extracellular vesicles. Advanced Sci. (2025) 12:2506121. doi: 10.1002/advs.202506121, PMID: 40650578 PMC12499395

[31] Li X Long Y Zhu Y Gu J Zhou P Miao C . Endothelial-derived CCL7 promotes macrophage polarization and aggravates septic acute lung injury via CCR1-mediated STAT1 succinylation. Advanced Sci. (2025) 12:2506209. doi: 10.1002/advs.202506209, PMID: 40755420 PMC12520477

[32] Deng C Xiao Y Zhao X Li H Chen Y Ai K . Sequential targeting chondroitin sulfate-bilirubin nanomedicine attenuates osteoarthritis via reprogramming lipid metabolism in M1 macrophages. Advanced Sci. (2025) 12:2411911. doi: 10.1002/advs.202411911, PMID: 39792653 PMC11884591

[33] Zhou J Li H Lu K . Selective autophagy of the immunoproteasomes suppresses innate inflammation. Autophagy. (2024) 20:2107–8. doi: 10.1080/15548627.2024.2353437, PMID: 38719780 PMC11346561

[34] Wang L Cui J . Palmitoylation promotes chaperone-mediated autophagic degradation of NLRP3 to modulate inflammation. Autophagy. (2023) 19:2821–3. doi: 10.1080/15548627.2023.2187957, PMID: 36927399 PMC10472840

[35] Yao X Rudensky E Martin PK Miller BM Vargas I Zwack EE . Heterozygosity for Crohn’s disease risk allele of ATG16L1 promotes unique protein interactions and protects against bacterial infection. Immunity. (2025) 58:1456–68.e5. doi: 10.1016/j.immuni.2025.04.023, PMID: 40373771 PMC12158642

[36] Gao H Wang L Lyu Y Jin H Lin Z Kang Y . The P2X7R/NLRP3 inflammasome axis suppresses enthesis regeneration through inflammatory and metabolic macrophage-stem cell cross-talk. Sci Adv. (2025) 11:adr4894. doi: 10.1126/sciadv.adr4894, PMID: 40279432 PMC12024643

[37] Ramos ELF Sousa Neto IVd Pinto AP Cintra DE Ropelle ER Pauli JR . Mechanisms underlying the interplay between autophagy and the inflammasome in age-related diseases: implications for exercise immunology. Ageing Res Rev. (2025) 110:102821. doi: 10.1016/j.arr.2025.102821, PMID: 40609647

[38] Molina-López C Hurtado-Navarro L García CJ Angosto-Bazarra D Vallejo F . Pathogenic NLRP3 mutants form constitutively active inflammasomes resulting in immune-metabolic limitation of IL-1β production. Nat Commun. (2024) 15:44990. doi: 10.1038/s41467-024-44990-0, PMID: 38321014 PMC10847128

[39] Humayun S Rjabovs V Justine EE Darko CNS Howlader MM Reile I . Immunomodulatory activity of red algal galactans and their partially depolymerized derivatives in RAW264.7 macrophages. Carbohydr Polymers. (2025) 347:122741. doi: 10.1016/j.carbpol.2024.122741, PMID: 39486970

[40] Bai T Bao S Li Y Hou X Pan S Wang H . The structural discrepancy between the ability of fructan and arabinogalactan to cure acute pharyngitis in Hosta plantaginea (Lam.) Aschers flowers. Carbohydr Polymers. (2025) 350:123059. doi: 10.1016/j.carbpol.2024.123059, PMID: 39647959

[41] Cao C Liao Y Yu Q Zhang D Huang J Su Y . Structural characterization of a galactoglucomannan with anti-neuroinflammatory activity from Ganoderma lucidum. Carbohydr Polymers. (2024) 334:122030. doi: 10.1016/j.carbpol.2024.122030, PMID: 38553228

[42] Yu R Zhang M Liao J Zhang Z Wu J Huang W . Structural characterization of two novel heteropolysaccharides from Catharanthus roseus and the evaluation of their immunological activities. (2024). doi: 10.2139/ssrn.4782166, PMID: 39567132

[43] Zhang W Liu W Leng F Shen M Xie J . Dietary non-starch plant polysaccharides: Multi-mechanisms for managing diabetic microvascular complications. Carbohydr Polymers. (2025) 368:124074. doi: 10.1016/j.carbpol.2025.124074, PMID: 40912791

[44] Zhu L Gong H Gan X Bu Y Liu Y Zhang T . Processing-structure-activity” relationships of polysaccharides in Chinese Materia Medica: A comprehensive review. Carbohydr Polymers. (2025) 358:123503. doi: 10.1016/j.carbpol.2025.123503, PMID: 40383564

[45] Qiu Z Chen L Hou X Sheng J Xu J Xu J . Toxoplasma gondii infection triggers ongoing inflammation mediated by increased intracellular Cl– concentration in airway epithelium. J Infection. (2023) 86:47–59. doi: 10.1016/j.jinf.2022.10.037, PMID: 36334726

[46] Xie L Chen H Zhang L Yang Y Zhou Y Ma Y . Suppressing MASH fibrotic progression by blocking succinate-GPR91 signaling in HSCs. Hepatology. (2025). doi: 10.1097/hep.0000000000001405, PMID: 40392081

[47] Cheng C Zhang J Li X Xue F Cao L Meng L . NPRC deletion mitigated atherosclerosis by inhibiting oxidative stress, inflammation and apoptosis in ApoE knockout mice. Signal Transduction Targeted Ther. (2023) 8:1560. doi: 10.1038/s41392-023-01560-y, PMID: 37553374 PMC10409771

[48] Peng Y Zhu X Yang G Zhang J Wang R Shen Y . Ultrasonic extraction of Moringa oleifera seeds polysaccharides: Optimization, purification, and anti-inflammatory activities. Int J Biol Macromolecules. (2024) 258:128833. doi: 10.1016/j.ijbiomac.2023.128833, PMID: 38128806

[49] He X Deng B Zhang C Zhang G Yang F Zhu D . HSPA1A inhibits pyroptosis and neuroinflammation after spinal cord injury via DUSP1 inhibition of the MAPK signaling pathway. Mol Med. (2025) 31:1086. doi: 10.1186/s10020-025-01086-9, PMID: 39924492 PMC11809008

[50] Xiao Y Yan Y Du J Feng X Zhang F Han X . Novel 2-phenyl-4H-chromen derivatives: synthesis and anti-inflammatory activity evaluation. J Enzyme Inhibition Medicinal Chem. (2022) 37:2589–97. doi: 10.1080/14756366.2022.2124983, PMID: 36128868 PMC9518258

[51] Wang Q Xian M Li C Mi L Ji G Wu Z . Protective effect of liquiritin against cisplatin-induced liver injury in mice through reduction of inflammation, oxidative stress, apoptosis and inhibition of p38 MAPK/p53 pathway based on network pharmacology and in experimental validation. (2025). doi: 10.2139/ssrn.5177525, PMID: 40912484

[52] Jiang Y Qi S Mao C . Polysaccharide nanoparticles as potential immune adjuvants: Mechanism and function. Acta Pharm Sin B. (2025) 15:1796–815. doi: 10.1016/j.apsb.2025.03.006, PMID: 40486863 PMC12137980

[53] Huang S Ding S Fan L . A review of the immunomodulatory activities of polysaccharides isolated from Inonotus obliquus. Int J Biol Macromolecules. (2012) 50:1183–7. doi: 10.1016/j.ijbiomac.2012.03.019, PMID: 22484729

[54] Zhang Y Lu J Li H Song H . Advances in dietary polysaccharides as hypoglycemic agents: mechanisms, structural characteristics, and innovative applications. Crit Rev Food Sci Nutr. (2023) 65:1383–403. doi: 10.1080/10408398.2023.2293254, PMID: 38095578

[55] Liu G Kamilijiang M Abuduwaili A Zang D Abudukelimu N Liu G . Isolation, structure elucidation, and biological activity of polysaccharides from Saussurea involucrata. Int J Biol Macromolecules. (2022) 222:154–66. doi: 10.1016/j.ijbiomac.2022.09.137, PMID: 36122780

[56] Liu W Kong Y Wang X Yang Y Yan Q Li Z . Therapeutic mechanisms of polysaccharides in the management of rheumatoid arthritis: a comprehensive review. Front Immunol. (2025) 16:1608909. doi: 10.3389/fimmu.2025.1608909, PMID: 40666523 PMC12259611

[57] Gao M Zhang W Ma Y Liu T Wang S Chen S . Bioactive polysaccharides prevent lipopolysaccharide-induced intestinal inflammation via immunomodulation, antioxidant activity, and microbiota regulation. Foods. (2025) 14:2575. doi: 10.3390/foods14152575, PMID: 40807512 PMC12346005

[58] Ye M Fan M Zhao Y Wang F Yang X Yao W . Astragalus membranaceus polysaccharides ameliorate dextran sulfate sodium-induced colitis via regulating TIGIT/CD155 signaling pathway and restoring Th17/Treg balance. Carbohydr Polymers. (2025) 367:124050. doi: 10.1016/j.carbpol.2025.124050, PMID: 40817502

[59] Wang M Yu A Hu W Zhang Z Wang Z Meng Y . Extraction, purification, structural characteristic, health benefit, and product application of the polysaccharides from bamboo shoot: A review. Int J Biol Macromolecules. (2024) 271:132581. doi: 10.1016/j.ijbiomac.2024.132581, PMID: 38797301

[60] Fang C Chen S Liao C Chen J Yen G . Fagopyrum esculentum polysaccharides mitigate obesity by reshaping gut microbiota and enhancing lipid metabolism in high-fat diet-fed mice. Int J Biol Macromolecules. (2025) 322:146668. doi: 10.1016/j.ijbiomac.2025.146668, PMID: 40780346

[61] Meng J Deng K Hu N Wang H . Nitraria tangutorum Bobr.-derived polysaccharides protect against LPS-induced lung injury. Int J Biol Macromolecules. (2021) 186:71–8. doi: 10.1016/j.ijbiomac.2021.06.181, PMID: 34216671

[62] Bi H Teng W Wang J Wang X Zhang Z Wang M . Extraction and purification, structural characteristics, pharmacological activities, structure-activity relationships, applications, and quality assessments of Prunella vulgaris L. polysaccharides: A review. Int J Biol Macromolecules. (2025) 306:141665. doi: 10.1016/j.ijbiomac.2025.141665, PMID: 40037438

[63] Lu M Chao C Hsu Y . Advanced culture strategy shows varying bioactivities of sulfated polysaccharides of Poria cocos. Int J Biol Macromolecules. (2023) 253:126669. doi: 10.1016/j.ijbiomac.2023.126669, PMID: 37660853

[64] He Y Gao W Zhang Y Sun M Kuang H Sun Y . Progress in the preparation, structure and bio-functionality of Dictyophora indusiata polysaccharides: A review. Int J Biol Macromolecules. (2024) 283:137519. doi: 10.1016/j.ijbiomac.2024.137519, PMID: 39577539

[65] Fu Y Wang Q Guo Y Koci M Lu Z Zeng X . Pleurotus eryngii polysaccharides alleviate aflatoxin B-induced liver inflammation in ducks involving in remodeling gut microbiota and regulating SCFAs transport via the gut-liver axis. Int J Biol Macromolecules. (2024) 271:132371. doi: 10.1016/j.ijbiomac.2024.132371, PMID: 38750861

[66] Zhang J Chen X Zhan Q Wang Y Meng K Hu Q . Studying on the structure-activity relationship of Flammulina velutipes polysaccharides via ultrasonic degradation: Insights into molecular weight, chain conformation, and anti-inflammatory activity. Int J Biol Macromolecules. (2025) 302:140480. doi: 10.1016/j.ijbiomac.2025.140480, PMID: 39889991

[67] Cao Y Gao J Zhang L Qin N Zhu B Xia X . Jellyfish skin polysaccharides enhance intestinal barrier function and modulate the gut microbiota in mice with DSS-induced colitis. Food Funct. (2021) 12:10121–35. doi: 10.1039/d1fo02001c, PMID: 34528649

[68] Zhu Z Han Y Ding Y Zhu B Song S Xiao H . Health effects of dietary sulfated polysaccharides from seafoods and their interaction with gut microbiota. Compr Rev Food Sci Food Safety. (2021) 20:2882–913. doi: 10.1111/1541-4337.12754, PMID: 33884748

[69] Kalita P Ahmed AB Sen S Chakraborty R . A comprehensive review on polysaccharides with hypolipidemic activity: Occurrence, chemistry and molecular mechanism. Int J Biol Macromolecules. (2022) 206:681–98. doi: 10.1016/j.ijbiomac.2022.02.189, PMID: 35247430

[70] Zheng C Li T Tang Y Lu T Wu M Sun J . Structural and functional investigation on stem and peel polysaccharides from different varieties of pitaya. Int J Biol Macromolecules. (2024) 259:129172. doi: 10.1016/j.ijbiomac.2023.129172, PMID: 38176496

[71] Ansari E Alvandi H Kianirad S Hatamian-Zarmi A Mokhtari-Hosseini ZB . Research progress on production and biomedical applications of Schizophyllan as a tailor-made polysaccharide: A review. Carbohydr Polymers. (2025) 348:122770. doi: 10.1016/j.carbpol.2024.122770, PMID: 39562055

[72] Wang X Zeng Z Lin Y Zhang Y Pan L . The application of Gracilaria lemaneiformis polysaccharide as a potential flavor improver and stabilizer for set yogurt. Int J Biol Macromolecules. (2025) 329:147692. doi: 10.1016/j.ijbiomac.2025.147692, PMID: 40957520

[73] Van DTT Hoang TLH Tran TVT Le TH Le LSS Nguyen QM . Structural Characterizations and *In Vitro* Bioactivities of a d-Fructose-Rich Polysaccharide from Gymnopetalum cochinchinense as a Potential Candidate for Alleviating Alzheimer’s Disease. ACS Omega. (2025) 10:40342–53. doi: 10.1021/acsomega.5c05628, PMID: 40949219 PMC12423878

[74] Cao W Liu Y Chen N Wang Y Nushrat YM Qiao S . Preparation, characterization, fermentation properties of pectin with specific structures, and the analysis of microbial enzymes and genes involved in their degradation. Carbohydr Polymers. (2025) 368:124162. doi: 10.1016/j.carbpol.2025.124162, PMID: 40947252

[75] He W Jiang H Rong M Li X Xu J Guo Y . A Fungal Polysaccharide from Trametes orientalis: Structural Elucidation and Multifaceted Antitumor Mechanisms via Metastasis, Angiogenesis, and Immunomodulation. Carbohydr Polymers. (2025) 368:124151. doi: 10.1016/j.carbpol.2025.124151, PMID: 40947190

[76] Huang H Wang Y Chen J Tan T Yang D . Ultrasound-Microwave Synergistic Extraction Enhances Bioactivities of Phyllanthus emblica L. Polysaccharides through Structure-Function Modulation. Ultrasonics Sonochemistry. (2025) 121:107564. doi: 10.1016/j.ultsonch.2025.107564, PMID: 40946471 PMC12504964

[77] Du X Wang J Gao L Zheng J Zhang L . Study on the structural characterization and biological activities of polysaccharides from Dictyophora rubrovolvata and its silver nanoparticles. Int J Biol Macromolecules. (2025) 328:147632. doi: 10.1016/j.ijbiomac.2025.147632, PMID: 40945821

[78] Zhang H Hasegawa Y . Protective effect of a highly enriched nacre-derived neutral polysaccharide fraction on D-galactose-induced pancreatic dysfunction. Molecules. (2025) 30:3555. doi: 10.3390/molecules30173555, PMID: 40942080 PMC12430628

[79] Zhang X An S Zhou L Chen C Yang X . Structural characterization and anti-gout activity of a novel acidic Sanghuangporus vaninii polysaccharide. Molecules. (2025) 30:3536. doi: 10.3390/molecules30173536, PMID: 40942068 PMC12429920

[80] Wei X Du P Luo Y Zhao Y Zhou X Chen G . Degraded polysaccharides from noni (Morinda citrifolia L.) juice mitigate glucose metabolism disorders by regulating PI3K/AKT-Nrf2-GSK3β Signaling pathways in HepG2 cells. Foods. (2025) 14:2989. doi: 10.3390/foods14172989, PMID: 40941105 PMC12427753

[81] Li M He R Yang C Chen H Wu C Zhang X . Response Surface Methodology Optimization Extraction of Polysaccharide from Lilium brownii F.E. Brown var. viridulum Baker/Longya Baihe and Its Biological Activities. Preparative Biochem Biotechnol. (2025), 1–15. doi: 10.1080/10826068.2025.2556871, PMID: 40932170

[82] Shen W Luo R Yang L Li Z Hou X Liu Z . Psyllium-derived, medium-molecular-weight arabinoxylan enables modulation of the gut microbiota and metabolites in type 2 diabetic mice. Int J Biol Macromolecules. (2025) 328:147363. doi: 10.1016/j.ijbiomac.2025.147363, PMID: 40921369

[83] Ren T Huang Y Han Y Li B Han L Li Y . Structure Analysis of an Acidic Polysaccharide Isolated from Epimedium koreanum Nakai and Its Improvement on Liver Oxidative Stress Injury in STZ-Induced Diabetic Rats via NRF2/HO-1 Pathways. Int J Biol Macromolecules. (2025) 328:147367. doi: 10.1016/j.ijbiomac.2025.147367, PMID: 40921367

[84] Zhang S Wu Z Zhang Q Zhao H Li J . Ultrasonic-assisted three-phase partitioning with deep eutectic solvents for extraction of Nostoc commune polysaccharides and their anti-ulcerative colitis activity. Int J Biol Macromolecules. (2025) 328:147484. doi: 10.1016/j.ijbiomac.2025.147484, PMID: 40921363

[85] Guo D Lei J Zhu H Mu W Cheng Y Guo Y . Ultrasound-microwave-enzyme synergistic extraction of Brassica rapa L. Polysaccharides: structural characterization, *in vitro* fecal fermentation dynamics, and gut microbiota modulation. Int J Biol Macromolecules. (2025) 327:147493. doi: 10.1016/j.ijbiomac.2025.147493, PMID: 40915454

[86] Yi Y Zhang C Li Q Fan X Wang Y . Formation of formaldehyde in dialdehyde polysaccharides tanning agent: effect of polysaccharide structural characteristic. Int J Biol Macromolecules. (2025) 327:147499. doi: 10.1016/j.ijbiomac.2025.147499, PMID: 40915446

[87] Gao X Wang L Niu J Li J Yu M Martins FS . Extraction, purification, structural characterization and immunomodulatory activity of a polysaccharide from Hirudo nipponica Whitman. Int J Biol Macromolecules. (2025) 327:147471. doi: 10.1016/j.ijbiomac.2025.147471, PMID: 40915443

[88] Li T Lai M Memtimin M Yao M Yang C Liu P . A novel acidic polysaccharide from Isatidis radix: structural characterization and immunoregulatory effect in Zebrafish. Int J Biol Macromolecules. (2025) 327:147470. doi: 10.1016/j.ijbiomac.2025.147470, PMID: 40914373

[89] Yang M Li X Li F Du X Wang T Gao Y . Ultrasound-assisted enzymatic extraction optimization of Cistanche deserticola polysaccharides, and combined analysis of the relationship between polysaccharide structure and activity by liquid chromatography and GC-MS. J Chromatogr A. (2025) 1761:466331. doi: 10.1016/j.chroma.2025.466331, PMID: 40902278

[90] Han X Ren X Zhang D Guo Q Li S Xiu Z . A novel polysaccharide in Polygonatum kingianum: structure elucidation, the activities of anti-inflammatory and the regulation of gut microbiota *in vitro*. Natural Products Bioprospecting. (2025) 15:542. doi: 10.1007/s13659-025-00542-7, PMID: 40892304 PMC12405078

[91] Kowlakuntla ER TCV . Purification of O-antigen for polysaccharide vaccine development against Salmonella paratyphi. Preparative Biochem Biotechnol. (2025), 1–9. doi: 10.1080/10826068.2025.2555304, PMID: 40891258

[92] Zhou Q Li X Ma X Gao Y Li Y Xie H . Structural Characterization of a Neutral Heteropolysaccharide from Pholidota chinensis Lindl. and Its Protective Effects Against Intestinal Inflammation via Barrier Enhancement and Gut Microbiota Modulation. Int J Biol Macromolecules. (2025) 325:147181. doi: 10.1016/j.ijbiomac.2025.147181, PMID: 40889656

[93] Zhong A Li Q Su H Huang L Zhou Q Wang X . Structural characterization and ameliorative effects of Mesona chinensis Benth polysaccharide against deoxynivalenol-induced oxidative stress in intestinal epithelial cells. Nutrients. (2025) 17:2592. doi: 10.3390/nu17162592, PMID: 40871619 PMC12389029

[94] Fan R Zhang W Wang L Fei T Xiao J Wang L . Structural characterization of a novel pectin polysaccharide from mango (Mangifera indica L.) peel and its regulatory effects on the gut microbiota in high-fat diet-induced obese mice. Foods. (2025) 14:2910. doi: 10.3390/foods14162910, PMID: 40870827 PMC12385833

[95] Zhang L Zhao S Wang J Zhang J Zheng T Li J . Enzymatic degradation, structural characterization, and *in vitro* antioxidant, hypoglycemic, and anti-inflammatory activities of Sanghuang vaninii polysaccharides. Int J Biol Macromolecules. (2025) 323:147114. doi: 10.1016/j.ijbiomac.2025.147114, PMID: 40865830

[96] Eltay EG Van Dyke T . Resolution of inflammation in oral diseases. Pharmacol Ther. (2023) 247:108453. doi: 10.1016/j.pharmthera.2023.108453, PMID: 37244405

[97] Kim S Han S Kim M Mony TJ Lee E Kim K . Essential oil exerts anti-inflammatory in LPS-stimulated inflammatory responses via inhibition of ERK/NF-κB signaling pathway and anti-atopic dermatitis-like effects in 2,4-dinitrochlorobezene-induced BALB/c mice. Antioxidants. (2021) 10:1941. doi: 10.3390/antiox10121941, PMID: 34943044 PMC8750489

[98] Li M Wen J Huang X Nie Q Wu X Ma W . Interaction between polysaccharides and toll-like receptor 4: Primary structural role, immune balance perspective, and 3D interaction model hypothesis. Food Chem. (2022) 374:131586. doi: 10.1016/j.foodchem.2021.131586, PMID: 34839969

[99] Yu J Zhou L Song H Huang Q Yu J Wang S . (-)-Epicatechin gallate blocked cellular foam formation in atherosclerosis by modulating CD36 expression and. Food Funct. (2023) 14:2444–58. doi: 10.1039/d2fo03218j, PMID: 36786689

[100] Kim M Kim JY Yang HS Choe J Hwang IG . Nepetoidin B from R. Br. Inhibits inflammation by modulating the NF-κB and Nrf2/HO-1 signaling pathways in macrophage cells. Antioxidants. (2021) 10:1208. doi: 10.3390/antiox10081208, PMID: 34439456 PMC8388923

[101] Zhou H Yu W Yan X Liang X Ma X Long J . Lactate-driven macrophage polarization in the inflammatory microenvironment alleviates intestinal inflammation. Front Immunol. (2022) 13:1013686. doi: 10.3389/fimmu.2022.1013686, PMID: 36330516 PMC9623299

[102] Li Y Liu Z Yan H Zhou T Zheng L Wen F . Polygonatum sibiricum polysaccharide ameliorates skeletal muscle aging and mitochondrial dysfunction via PI3K/Akt/mTOR signaling pathway. (2024). doi: 10.2139/ssrn.4923691, PMID: 39674120

[103] Qiu W Chao C Hsu Y Lu M . Anti-inflammatory potential of low-molecular-weight and high-sulfation-degree sulfated polysaccharides extracted from Antrodia cinnamomea. Int J Biol Macromolecules. (2024) 277:134360. doi: 10.1016/j.ijbiomac.2024.134360, PMID: 39094855

[104] Bhattacharjee A Singh N Kumar P Katti DS . Sulfated carboxymethylcellulose mediated enhancement of Timp3 efficacy synergistically attenuates osteoarthritis through inhibition of NFκB and JNK. Carbohydr Polymers. (2023) 316:121061. doi: 10.1016/j.carbpol.2023.121061, PMID: 37321710

[105] Esposito F Vessella G Sinquin C Traboni S Iadonisi A Colliec-Jouault S . Glycosaminoglycan-like sulfated polysaccharides from Vibrio diabolicus bacterium: Semi-synthesis and characterization. Carbohydr Polymers. (2022) 283:119054. doi: 10.1016/j.carbpol.2021.119054, PMID: 35153009

[106] Wang C Wang P Fu J Yang Z Du H Zhang M . Pinus massoniana pollen polysaccharides alleviate LPS-induced myocardial injury through p110β-mediated inhibition of the PI3K/AKT/NFκB pathway. Int J Biol Macromolecules. (2024) 283:137713. doi: 10.1016/j.ijbiomac.2024.137713, PMID: 39551317

[107] Yu S Li D Shi A Long Y Deng J Ma Y . Multidrug-loaded liposomes prevent ischemic stroke through intranasal administration. Biomedicine Pharmacother. (2023) 162:114542. doi: 10.1016/j.biopha.2023.114542, PMID: 36989725

[108] Xing H Bai X Pei X Zhang Y Zhang X Chen S . Synergistic anti-oxidative/anti-inflammatory treatment for acute lung injury with selenium based chlorogenic acid nanoparticles through modulating Mapk8ip1/MAPK and Itga2b/PI3k-AKT axis. J Nanobiotechnol. (2025) 23:3114. doi: 10.1186/s12951-025-03114-6, PMID: 39849453 PMC11756189

[109] Liu X Wang N Cai T Chen H Dai C Zhu W . Exploring the material basis and mechanism of Astilbe chinensis for acute lung injury via integrated network pharmacology and experimental validation. J Ethnopharmacol. (2025) 353:120438. doi: 10.1016/j.jep.2025.120438, PMID: 40850661

[110] Alias AHD Shafie MH . Toward sustainable obesity management: A review of extraction, properties, and structure-activity relationships of antioxidant and anti-obesity polysaccharides. Int J Biol Macromolecules. (2025) 321:146171. doi: 10.1016/j.ijbiomac.2025.146171, PMID: 40692053

[111] Li Q Yang F Hou R Huang T Hao Z . Post-screening characterization of an acidic polysaccharide from with potent anti-inflammatory properties. Food Funct. (2020) 11:7576–83. doi: 10.1039/d0fo01367f, PMID: 32821898

[112] Zhao C Li H Gao C Tian H Guo Y Liu G . Moringa oleifera leaf polysaccharide regulates fecal microbiota and colonic transcriptome in calves. Int J Biol Macromolecules. (2023) 253:127108. doi: 10.1016/j.ijbiomac.2023.127108, PMID: 37776927

[113] Anti-inflammation mechanisms of flavones are highly sensitive to the position isomers of flavonoids: acacetin vs biochanin A. J Agric Food Chem. (2024) 72(37):11121–30. doi: 10.1021/acs.jafc.4c05060.s001, PMID: 39287184

[114] Zou Y Wu S Zhang W Zhang W Shen X Qu X . Fucoidan improves tumour control and liver function in TACE for unresectable hepatocellular carcinoma: A randomised trial. Liver Int. (2025) 45:70347. doi: 10.1111/liv.70347, PMID: 40960276 PMC12442522

[115] Li X Zhao H Liu K Liu M Qing X Yu W . Structural determination, immunomodulatory activity, and antitumor activity of a low-molecular-weight polysaccharide extracted from Lepista sordida. Int J Biol Macromolecules. (2025) 307:141973. doi: 10.1016/j.ijbiomac.2025.141973, PMID: 40081683

[116] Wu L Zhou Z Sathe D Zhou J Reich S . Precision native polysaccharides from living polymerization of anhydrosugars. (2023). doi: 10.26434/chemrxiv-2023-rht0l, PMID: 37106096

[117] Esposito F Laezza A Gargiulo V Traboni S Iadonisi A La Gatta A . Multi-step strategies toward regioselectively sulfated M-rich alginates. Biomacromolecules. (2023) 24:2522–31. doi: 10.1021/acs.biomac.3c00045, PMID: 37116076 PMC10265665

[118] Li J Wang Y Shen Z Zou Q Lin X Wang X . Recent developments on natural polysaccharides as potential anti-gastric cancer substance: Structural feature and bioactivity. Int J Biol Macromolecules. (2023) 232:123390. doi: 10.1016/j.ijbiomac.2023.123390, PMID: 36706878

[119] Li D Chen M Meng X Sun Y Liu R Sun T . Extraction, purification, structural characteristics, bioactivity and potential applications of polysaccharides from Avena sativa L.: A review. Int J Biol Macromolecules. (2024) 265:130891. doi: 10.1016/j.ijbiomac.2024.130891, PMID: 38493821

[120] Mao S Zhang X Yang M Duan J Xiao P . A comprehensive review on the antitumor mechanisms of polysaccharides and their structure-activity relationships-Current insights and future directions. Int J Biol Macromolecules. (2025) 319:145355. doi: 10.1016/j.ijbiomac.2025.145355, PMID: 40562142

[121] Guo X Xin Q Wei P Hua Y Zhang Y Su Z . Antioxidant and anti-aging activities of Longan crude and purified polysaccharide (LP-A) in nematode Caenorhabditis elegans. Int J Biol Macromolecules. (2024) 267:131634. doi: 10.1016/j.ijbiomac.2024.131634, PMID: 38636747

[122] Qiao M Xue T Zhu Y Yang J Hu J . Polysaccharides from Cistanche deserticola mitigate inflammatory bowel disease via modulating intestinal microbiota and SRC/EGFR/PI3K/AKT signaling pathways. Int J Biol Macromolecules. (2025) 308:142452. doi: 10.1016/j.ijbiomac.2025.142452, PMID: 40139591

[123] Tang X Mo D Jiang N Kou Y Zhang Z Peng R . Polysaccharides from maggot extracts suppressed colorectal cancer progression by inducing ferroptosis via HMOX1/GPX4 signaling pathway. Int J Biol Macromolecules. (2025) 296:139734. doi: 10.1016/j.ijbiomac.2025.139734, PMID: 39798758

[124] Saeid A Dave D Shahidi F . Polysaccharides from echinoderms: unlocking health benefits and food applications - a review. Food Funct. (2025) 16:5679–704. doi: 10.1039/d5fo02177d, PMID: 40552666

[125] Deng Y Song L Huang J Zhou W Liu Y Lu X . Astragalus polysaccharides ameliorates experimental colitis by regulating memory B cells metabolism. Chemico-Biological Interactions. (2024) 394:110969. doi: 10.1016/j.cbi.2024.110969, PMID: 38522565

[126] Wang H Zhu W Hong Y Wei W Zheng N He X . Astragalus polysaccharide attenuates chemotherapy-induced immune injury by modulating gut microbiota and polyunsaturated fatty acid metabolism. Phytomedicine. (2024) 128:155492. doi: 10.1016/j.phymed.2024.155492, PMID: 38479258

[127] Li N Zhang Y Han M Liu T Wu J Xiong Y . Self-adjuvant Astragalus polysaccharide-based nanovaccines for enhanced tumor immunotherapy: a novel delivery system candidate for tumor vaccines. Sci China Life Sci. (2023) 67:680–97. doi: 10.1007/s11427-023-2465-x, PMID: 38206438

[128] Zhou F Lu Y Sun T Sun L Wang B Lu J . Antitumor effects of polysaccharides from Tetrastigma hemsleyanum Diels et Gilg via regulation of intestinal flora and enhancing immunomodulatory effects *in vivo*. Front Immunol. (2022) 13:1009530. doi: 10.3389/fimmu.2022.1009530, PMID: 36389762 PMC9650377

[129] Fu S Bao X Wang Z Tang Y Wu Q Zhu B . Antipyretic effect of inhaled Tetrastigma hemsleyanum polysaccharide on substance and energy metabolism in yeast-induced pyrexia mice via TLR4/NF-κb signaling pathway. J Ethnopharmacol. (2024) 323:117732. doi: 10.1016/j.jep.2024.117732, PMID: 38218501

[130] Płóciennikowska A Hromada-Judycka A Borzęcka K Kwiatkowska K . Co-operation of TLR4 and raft proteins in LPS-induced pro-inflammatory signaling. Cell Mol Life Sci. (2014) 72:557–81. doi: 10.1007/s00018-014-1762-5, PMID: 25332099 PMC4293489

[131] Ciesielska A Matyjek M Kwiatkowska K . TLR4 and CD14 trafficking and its influence on LPS-induced pro-inflammatory signaling. Cell Mol Life Sci. (2020) 78:1233–61. doi: 10.1007/s00018-020-03656-y, PMID: 33057840 PMC7904555

[132] Albensi BC . What is nuclear factor kappa B (NF-κB) doing in and to the mitochondrion? Front Cell Dev Biol. (2019) 7:154. doi: 10.3389/fcell.2019.00154, PMID: 31448275 PMC6692429

[133] Hui H Wang Z Zhao X Xu L Yin L Wang F . Gut microbiome-based thiamine metabolism contributes to the protective effect of one acidic polysaccharide from Selaginella uncinata (Desv.) Spring against inflammatory bowel disease. J Pharm Analysis. (2024) 14:177–95. doi: 10.1016/j.jpha.2023.08.003, PMID: 38464781 PMC10921243

[134] Zhan X Li J Zhou T . Targeting Nrf2-mediated oxidative stress response signaling pathways as new therapeutic strategy for pituitary adenomas. Front Pharmacol. (2021) 12:565748. doi: 10.3389/fphar.2021.565748, PMID: 33841137 PMC8024532

[135] Chen B Lu Y Chen Y Cheng J . The role of Nrf2 in oxidative stress-induced endothelial injuries. J Endocrinol. (2015) 225:R83–99. doi: 10.1530/joe-14-0662, PMID: 25918130

[136] Kathem SH Nasrawi YS Mutlag SH Nauli SM . Limonene exerts anti-inflammatory effect on LPS-induced jejunal injury in mice by inhibiting NF-κB/AP-1 pathway. Biomolecules. (2024) 14:334. doi: 10.3390/biom14030334, PMID: 38540754 PMC10968638

[137] Wusiman A Jiang W Yu L Zhu T He J Liu Z . Cationic polymer-modified Alhagi honey polysaccharide PLGA nanoparticles as an adjuvant to induce strong and long-lasting immune responses. Int J Biol Macromolecules. (2021) 177:370–82. doi: 10.1016/j.ijbiomac.2021.02.130, PMID: 33621572

[138] Cai G Wusiman A Gu P Mao N Xu S Zhu T . Supplementation of Alhagi honey polysaccharides contributes to the improvement of the intestinal immunity regulating the structure of intestinal flora in mice. Food Funct. (2021) 12:9693–707. doi: 10.1039/d1fo01860d, PMID: 34664596

[139] Sasaki K Rooge S Gunewardena S Hintz JA Ghosh P Pulido Ruiz IA . Kupffer cells: inflammation pathways and cell-cell interactions in alcohol-associated liver disease. Hepatology. (2024) 81:870–87. doi: 10.1097/hep.0000000000000918, PMID: 38687563 PMC11616785

[140] Zhuang S Yu R Zhong J Liu P Liu Z . Rhein from Rheum rhabarbarum Inhibits Hydrogen-Peroxide-Induced Oxidative Stress in Intestinal Epithelial Cells Partly through PI3K/Akt-Mediated Nrf2/HO-1 Pathways. J Agric Food Chem. (2019) 67:2519–29. doi: 10.1021/acs.jafc.9b00037, PMID: 30779558

[141] Song J Zhao X Bo J Lv Z Li G Chen Y . A polysaccharide from Alhagi honey protects the intestinal barrier and regulates the Nrf2/HO-1-TLR4/MAPK signaling pathway to treat alcoholic liver disease in mice. J Ethnopharmacol. (2024) 321:117552. doi: 10.1016/j.jep.2023.117552, PMID: 38072293

[142] Ávila-Gálvez MÁ Giménez-Bastida JA Karadeniz B Romero-Reyes S Espín JC . Polyphenolic characterization and anti-inflammatory effect of *in vitro* digested extracts of eChinacea purpurea L. Plant parts in an inflammatory model of human colon cells. Int J Mol Sci. (2024) 25:1744. doi: 10.3390/ijms25031744, PMID: 38339018 PMC10855148

[143] Hsu J Yang C Chen J . Antioxidant, anti-α-glucosidase, antityrosinase, and anti-inflammatory activities of bioactive components from Morus alba. Antioxidants. (2022) 11:2222. doi: 10.3390/antiox11112222, PMID: 36421408 PMC9686747

[144] Li R Xue Z Jia Y Wang Y Li S Zhou J . Polysaccharides from mulberry (Morus alba L.) leaf prevents obesity by inhibiting pancreatic lipase in high-fat diet induced mice. Int J Biol Macromolecules. (2021) 192:452–60. doi: 10.1016/j.ijbiomac.2021.10.010, PMID: 34634334

[145] Zhang Y Li L Chai T Xu H Du H Jiang Y . Mulberry leaf multi-components exert hypoglycemic effects through regulation of the PI-3K/Akt insulin signaling pathway in type 2 diabetic rats. J Ethnopharmacol. (2024) 319:117307. doi: 10.1016/j.jep.2023.117307, PMID: 37939911

[146] Zheng Y Chen Q Yang H Zhao J Ren L Wu Y . Modulation of gut microbiota by crude mulberry polysaccharide attenuates knee osteoarthritis progression in rats. Int J Biol Macromolecules. (2024) 262:129936. doi: 10.1016/j.ijbiomac.2024.129936, PMID: 38309391

[147] Fu M Wang J Xu D Cao N Li W Li F . Polysaccharide of Atractylodes macrocephala Koidz alleviates LPS-induced proliferation, differentiation inhibition and excessive apoptosis in chicken embryonic myogenic cells. Veterinary Med Sci. (2024) 10:1412. doi: 10.1002/vms3.1412, PMID: 38504633 PMC10951630

[148] Zhao T Wang C Liu Y Li B Shao M Zhao W . The role of polysaccharides in immune regulation through gut microbiota: mechanisms and implications. Front Immunol. (2025) 16:1555414. doi: 10.3389/fimmu.2025.1555414, PMID: 40230839 PMC11994737

[149] Rajendran K Karthikeyan A Krishnan UM . Emerging trends in nano-bioactive-mediated mitochondria-targeted therapeutic stratagems using polysaccharides, proteins and lipidic carriers. Int J Biol Macromolecules. (2022) 208:627–41. doi: 10.1016/j.ijbiomac.2022.03.121, PMID: 35341885

[150] Gunawan M Boonkanokwong V . Current applications of solid lipid nanoparticles and nanostructured lipid carriers as vehicles in oral delivery systems for antioxidant nutraceuticals: A review. Colloids Surfaces B: Biointerfaces. (2024) 233:113608. doi: 10.1016/j.colsurfb.2023.113608, PMID: 37925866

[151] Rosales TKO da Silva FFA Bernardes ES Paulo Fabi J . Plant-derived polyphenolic compounds: nanodelivery through polysaccharide-based systems to improve the biological properties. Crit Rev Food Sci Nutr. (2023) 64:11894–918. doi: 10.1080/10408398.2023.2245038, PMID: 37585699

[152] Kumar D Pandey S Shivhare B Bala M Kumar M Kumar P . Natural polysaccharide-based nanodrug delivery systems for targeted treatment of rheumatoid arthritis: A review. Int J Biol Macromolecules. (2025) 310:143408. doi: 10.1016/j.ijbiomac.2025.143408, PMID: 40274161

[153] Yang B Xu Y Zhang W Zhu D Huang B Yang Y . Oral absorption mechanisms of polysaccharides and potential as carriers for the construction of nano-delivery systems: A review. Int J Biol Macromolecules. (2025) 310:143184. doi: 10.1016/j.ijbiomac.2025.143184, PMID: 40253019

[154] Guo X Liu H Hou R Chen G Xiao H Liu L . Design strategies of polysaccharide, protein and lipid-based nano-delivery systems in improving the bioavailability of polyphenols and regulating gut homeostasis. Int J Biol Macromolecules. (2024) 283:137463. doi: 10.1016/j.ijbiomac.2024.137463, PMID: 39547604

[155] Guo Z Tang S Nie K Liu J Hu C . Studies on absorption mechanism and pharmacokinetic properties of albendazole-bile acid conjugate: *In vivo* and *in vitro*. Biomedicine Pharmacother. (2024) 179:117400. doi: 10.1016/j.biopha.2024.117400, PMID: 39243427

[156] Allawadhi P Singh V Govindaraj K Khurana I Sarode LP Navik U . Biomedical applications of polysaccharide nanoparticles for chronic inflammatory disorders: Focus on rheumatoid arthritis, diabetes and organ fibrosis. Carbohydr Polymers. (2022) 281:118923. doi: 10.1016/j.carbpol.2021.118923, PMID: 35074100

[157] Liu T Fan S Meng P Ma M Wang Y Han J . Dietary dihydroquercetin alleviated colitis via the short-chain fatty acids/miR-10a-5p/PI3K-Akt signaling pathway. J Agric Food Chem. (2024) 72:23211–23. doi: 10.1021/acs.jafc.4c03278, PMID: 39393822

[158] The Distinct Properties of Polysaccharide Nanoparticles Tune Immune Responses against mRNA Antigen via Stimulator of Interferon Genes-Mediated Autophagy and Inflammasome. ACS Nano. (2023) 17:21782–98. doi: 10.1021/acsnano.3c07632.s001, PMID: 37922196

[159] Teunissen AJP Burnett ME Prévot G Klein ED Bivona D Mulder WJM . Embracing nanomaterials’ interactions with the innate immune system. WIREs Nanomedicine Nanobiotechnol. (2021) 13:1719. doi: 10.1002/wnan.1719, PMID: 33847441 PMC8511354

[160] Liu X Ou X Zhang T Li X Qiao Q Jia L . *In situ* neutrophil apoptosis and macrophage efferocytosis mediated by Glycyrrhiza protein nanoparticles for acute inflammation therapy. J Controlled Release. (2024) 369:215–30. doi: 10.1016/j.jconrel.2024.03.029, PMID: 38508529

[161] Xu Z Mao X Lu X Shi P Ye J Yang X . Dual-targeting nanovesicles carrying CSF1/CD47 identified from single-cell transcriptomics of innate immune cells in heart transplant for alleviating acute rejection. Advanced Healthcare Materials. (2023) 13:2302443. doi: 10.1002/adhm.202302443, PMID: 37962054

[162] Noh JY Han HW Kim DM Giles ED Farnell YZ Wright GA . Innate immunity in peripheral tissues is differentially impaired under normal and endotoxic conditions in aging. Front Immunol. (2024) 15:1357444. doi: 10.3389/fimmu.2024.1357444, PMID: 39221237 PMC11361940

[163] Li L Luo J Wang D Chang Y Duan C Zuo D . Fucoidan from Fucus vesiculosus Alleviates MetALD via Promoting HIF-1α Ubiquitination to Suppress Peripheral Monocyte Infiltration. Front Pharmacol. (2025) 16:1617175. doi: 10.3389/fphar.2025.1617175, PMID: 40894203 PMC12394499

[164] Guo Y Xu J Qi Y Zhou F Zhou M Zhu B . Tetrastigma hemsleyanum polysaccharide mitigates macrophage pyroptosis and prevents acute lung injury by regulating NLRP3/Caspase-1/GSDMD signaling pathways. Int J Biol Macromolecules. (2025) 323:147192. doi: 10.1016/j.ijbiomac.2025.147192, PMID: 40882722

[165] Afrin S Siddiqua Prova O Qureshi AT Ishaq MW Callmann CE Rizwan M . Injectable and self-healing fucoidan hydrogel: A natural anti-inflammatory biomaterial. Biomaterials. (2026) 326:123649. doi: 10.1016/j.biomaterials.2025.123649, PMID: 40882322 PMC13482689

[166] Fan Y Yang C Zhao Y Han X Ji H Ren Z . Regulatory effects of Codonopsis pilosula alkali-extracted polysaccharide-induced intestinal lactobacillus enrichment on peripheral blood proteomics in tumor-bearing mice. Microorganisms. (2025) 13:1750. doi: 10.3390/microorganisms13081750, PMID: 40871255 PMC12388226

[167] Wang R Qi Y Liu W Cheng Z Li W Zhu H . Multifunctional Gastrodia elata Polysaccharide-Based Triple-Network Hydrogel Promotes Staphylococcus aureus-Infected Diabetes Wound Healing. Carbohydr Polymers. (2025) 367:123983. doi: 10.1016/j.carbpol.2025.123983, PMID: 40817529

[168] Chen X Yang S Long X Li W Li B Fu C . Polysaccharide of Atractylodes macrocephala Koidz Alleviates LPS-Induced Inflammatory Liver Injury by Reducing Pyroptosis of Macrophage via Regulating lncRNA GAS5/miR-223-3p/NLRP3 Axis. Front Pharmacol. (2025) 16:1593689. doi: 10.3389/fphar.2025.1593689, PMID: 40799819 PMC12339467

[169] Zhang W Yuan S Chen R Li P Huang Q Zhou Z . Exopolysaccharide from endophytic fungi of Cinnamomum burmannii leaves: structural characterization and hepatoprotective effects. Int J Biol Macromolecules. (2025) 322:146703. doi: 10.1016/j.ijbiomac.2025.146703, PMID: 40784382

[170] Burkhart Colorado AS Nusbacher NM O’Connor J Marden T Higgins J Neff CP . The impact of western versus agrarian diet consumption on gut microbiome composition and immune dysfunction in people living with HIV in rural and urban Zimbabwe. (2025). doi: 10.1101/2025.07.18.665619, PMID: 42121284 PMC13339530

[171] Chen Y Li X Li Q Pan L Luo J Zha X . Development of chondroitin sulfate-modified quinoa protein isolate-dihydromyricetin composite nanoparticles: focus on naringenin delivery, cell cytoprotective ability and anti-inflammatory effects. Food Chem. (2025) 493:145761. doi: 10.1016/j.foodchem.2025.145761, PMID: 40768976

[172] Zhang Y . Fucoidan as a therapeutic agent for ulcerative colitis: mechanisms of action and modulation of the gut microbiota. Front Cell Infection Microbiol. (2025) 15:1626614. doi: 10.3389/fcimb.2025.1626614, PMID: 40708752 PMC12286961

[173] Yu Y Kong L Guo R Zhang Y Li S Zhang F . Engineered Panax notoginseng Polysaccharide Micelles Inhibit Macrophage Polarization and Delay the Progression of Rheumatoid Arthritis via JAK2-STAT3 Signaling Pathway. J Nanobiotechnol. (2025) 23:3576. doi: 10.1186/s12951-025-03576-8, PMID: 40660236 PMC12261558

[174] Li Y Liu L Huang X Huang H Jiang B Guo R . Probiotic fermentation of polygonatum plant polysaccharides converting fructans to glucans with enhanced anti-obesity activity. Int J Biol Macromolecules. (2025) 320:145871. doi: 10.1016/j.ijbiomac.2025.145871, PMID: 40659278

[175] Li X Ma K Tian T Pang H Liu T Li M . Methylmercury induces inflammatory response and autophagy in microglia through the activation of NLRP3 inflammasome. Environ Int. (2024) 186:108631. doi: 10.1016/j.envint.2024.108631, PMID: 38588609

[176] Li YC Hao JC Shang B Zhao C Wang LJ Yang KL . Neuroprotective effects of aucubin on hydrogen peroxide-induced toxicity in human neuroblastoma SH-SY5Y cells via the Nrf2/HO-1 pathway. Phytomedicine. (2021) 87:153577. doi: 10.1016/j.phymed.2021.153577, PMID: 33994055

[177] Li G Tang J Tan W Zhang T Zeng D Zhao S . The anti-hepatocellular carcinoma effects of polysaccharides from Pleurotus eryngii by regulating macrophage polarization through the MAPK/NF-κB signaling pathway. Food Funct. (2023) 14:3155–68. doi: 10.1039/d2fo02191a, PMID: 36883482

[178] Meng X Wei Q Wang S Liang S Wang D Kuang H . Anti-inflammatory effect of polysaccharides from Sambucus williamsii Hance roots in lipopolysaccharide-stimulated RAW264.7 macrophages and acute lung injury in mice. Int J Biol Macromolecules. (2025) 306:141368. doi: 10.1016/j.ijbiomac.2025.141368, PMID: 39988171

[179] Wang C Xu T Lachance BB Zhong X Shen G Xu T . Critical roles of sphingosine kinase 1 in the regulation of neuroinflammation and neuronal injury after spinal cord injury. J Neuroinflamm. (2021) 18:2092. doi: 10.1186/s12974-021-02092-4, PMID: 33602274 PMC7893778

[180] Huang C Wang X Zhang W Liu M Xie R Zheng H . Quercetin alleviates pyroptosis and necroptosis triggered on by DEHP exposure in bursa of fabricius in chicken by the ROS/MAPK/NF-κB pathway. J Agric Food Chem. (2025) 73:9337–47. doi: 10.1021/acs.jafc.5c00224, PMID: 40176274

[181] Ma C Zhang L Huang Q Deng Q Huang F Xu J . Canolol alleviates ethanol-induced gastric ulcer by inhibiting p38 MAPK/NF-κB/NLRP3 pathway. J Agric Food Chem. (2025) 73:9103–11. doi: 10.1021/acs.jafc.5c00621, PMID: 40179001

[182] Luo W Bai L Zhang J Li Z Liu Y Tang X . Polysaccharides-based nanocarriers enhance the anti-inflammatory effect of curcumin. Carbohydr Polymers. (2023) 311:120718. doi: 10.1016/j.carbpol.2023.120718, PMID: 37028867

[183] Jiang Y Yan C Li M Chen S Chen Z Yang L . Delivery of natural products via polysaccharide-based nanocarriers for cancer therapy: A review on recent advances and future challenges. Int J Biol Macromolecules. (2024) 278:135072. doi: 10.1016/j.ijbiomac.2024.135072, PMID: 39191341

[184] Yu L Xia D Chen Y Miao Y Xu R Pan Y . Novel 3,4-dihydronaphthalen-1(2H)-one derivatives promote apoptosis and inhibit migration of hepatocellular carcinoma cells via inhibition of NF-κB and MAPK signaling pathways. Eur J Medicinal Chem. (2025) 296:117898. doi: 10.1016/j.ejmech.2025.117898, PMID: 40561647

[185] Huang R Hou L Zhai X Ruan Z Sun W Zhang D . 2,5-hexanedione induces NLRP3 inflammasome activation and neurotoxicity through NADPH oxidase-dependent pathway. Free Radical Biol Med. (2021) 162:561–70. doi: 10.1016/j.freeradbiomed.2020.11.013, PMID: 33212186

[186] Probing the structural elements of polysaccharide adjuvants for enhancing respiratory mucosal response: from surmounting multi-obstacles to eliciting cascade immunity. ACS Nano. (2025) 19(11):11012–28. doi: 10.1021/acsnano.4c16788.s001, PMID: 40063734

[187] Cui Z Wang H Qin L Yuan Y Xue J An Y . Natural polysaccharide-based nano-drug delivery systems: Innovative strategies and research advances in cancer therapy - A review. Int J Biol Macromolecules. (2025) 321:146081. doi: 10.1016/j.ijbiomac.2025.146081, PMID: 40680959

[188] Hadji H Bouchemal K . Advances in the treatment of inflammatory bowel disease: Focus on polysaccharide nanoparticulate drug delivery systems. Advanced Drug Delivery Rev. (2022) 181:114101. doi: 10.1016/j.addr.2021.114101, PMID: 34999122

[189] Wang B Lin Y Zhou M Fu S Zhu B Chen Y . Polysaccharides from Tetrastigma hemsleyanum Diels et Gilg alleviate dry yeast-induced fever in rats by inhibiting the TLR4/MyD88/NF-κB signaling pathway. Biomedicine Pharmacother. (2022) 155:113755. doi: 10.1016/j.biopha.2022.113755, PMID: 36182735

[190] Song X Wang W Liu L Zhao Z Shen X Zhou L . Polysaccharides from Selaginella uncinata (Desv.) Spring alleviate DSS-induced colitis by regulating NF-κB/Nrf2/COX-2 pathway and modulating gut microbiota. Molecules. (2024) 29:2154. doi: 10.3390/molecules29092154, PMID: 38731645 PMC11085930

[191] Wu M Wang C Mai C Chen J Lai X He L . A novel acidic polysaccharide from Tamarix chinensis Lour ameliorates LPS-induced acute lung injury by inhibiting complement activation and inflammation. J Funct Foods. (2019) 61:103460. doi: 10.1016/j.jff.2019.103460

[192] Wu M Luo X Xu X Wei W Yu M Jiang N . Antioxidant, anti-inflammatory, and immunomodulatory activities of a polysaccharide from camelthorn honey. J Traditional Chin Med. (2014) 34:733–40. doi: 10.1016/s0254-6272(15)30089-3, PMID: 25618979

[193] Shi Q Lang W Wang S Li G Bai X Yan X . EChinacea purpurea polysaccharides protect against LPS-induced acute kidney injury by inhibiting oxidative stress and inflammation via MAPK signaling pathway. Int J Mol Med. (2020) 47:243–55. doi: 10.3892/ijmm.2020.4769, PMID: 33416087 PMC7723497

[194] Li P Xia Q Zhang H Hong R Wang Y Huang Y . Targeting macrophage glucose metabolism: polysaccharide-iron nanozyme-mediated reactive oxygen species/iron homeostasis restoration ameliorates inflammatory bowel disease with anemia comorbidity. J Colloid Interface Science. (2025) 700:138357. doi: 10.1016/j.jcis.2025.138357, PMID: 40633358

[195] Gao Z Liu X Yu J Li Z Shi H Zhang G . Structural basis of immunomodulation by edible fungal polysaccharides: from molecular characteristics to action mechanisms. Carbohydr Res. (2025) 555:109591. doi: 10.1016/j.carres.2025.109591, PMID: 40592243

[196] Generalov E Grigoryan I Minaichev V Sinitsyna O Yakovenko L Sinitsyn A . Anti-inflammatory effects of Solanum tuberosum L. Polysaccharide and its limited gene expression profile. Int J Mol Sci. (2025) 26:5562. doi: 10.3390/ijms26125562, PMID: 40565025 PMC12192999

[197] Zhao Y Sun Q Zhao T Miao C Pei H . Spirulina subsalsa polysaccharide: self-assembling hydrogel material for immunotherapy applications. Bioresource Technol. (2025) 435:132830. doi: 10.1016/j.biortech.2025.132830, PMID: 40532830

[198] Li H Wang F Di Y Jiang P Wang G . An alkali-extracted acidic heteropolysaccharide from Portulaca oleracea L. Ameliorates acute lung injury in septic mice by inhibiting macrophage pyroptosis. Int J Biol Macromolecules. (2025) 318:145139. doi: 10.1016/j.ijbiomac.2025.145139, PMID: 40505906

[199] Chen Y Lin Y Liu Z Zhang Y Hu Z Ai M . ROS-responsive adaptive injectable hydrogel promoting inflammatory mastoid bone repair through efficient sterilization and regulating oxidative stress and macrophage phenotype. Materials Today Bio. (2025) 32:101856. doi: 10.1016/j.mtbio.2025.101856, PMID: 40496721 PMC12148671

[200] Huo J Li M Wei J Wang Y Hao W Sun W . RNA-seq based elucidation of mechanism underlying the protective effect of Huangshui polysaccharide on intestinal barrier injury in Caco-2 cells. Food Res Int. (2022) 162:112175. doi: 10.1016/j.foodres.2022.112175, PMID: 36461372

[201] Yan W Luo J Yu Z Xu B . A critical review on intestinal mucosal barrier protection effects of dietary polysaccharides. Food Funct. (2024) 15:481–92. doi: 10.1039/d3fo03412g, PMID: 38197139

[202] Zong X Xiao X Kai L Cheng Y Fu J Xu W . Atractylodis macrocephalae polysaccharides protect against DSS-induced intestinal injury through a novel lncRNA ITSN1-OT1. Int J Biol Macromolecules. (2021) 167:76–84. doi: 10.1016/j.ijbiomac.2020.11.144, PMID: 33248053

[203] Wu Y Li A Liu H Zhang Z Zhang C Ma C . HNU082 alleviates dextran sulfate sodium-induced ulcerative colitis in mice through regulating gut microbiome. Food Funct. (2022) 13:10171–85. doi: 10.1039/d2fo02303b, PMID: 36111438

[204] Li J Wei Y Liu C Guo X Liu Z Zhang L . 2’-Fucosyllactose restores the intestinal mucosal barrier in ulcerative colitis by inhibiting STAT3 palmitoylation and phosphorylation. Clin Nutr. (2024) 43:380–94. doi: 10.1016/j.clnu.2023.12.011, PMID: 38150914

[205] Zongo AW Zogona D Youssef M Ye S Zhan F Li J . Plantago asiatica seed polysaccharides attenuate inflammation-induced intestinal epithelial barrier dysfunction in a Caco-2 and RAW264.7 macrophage co-culture model by inhibiting the NF-κB/MLCK pathway. Food Funct. (2022) 13:11676–89. doi: 10.1039/d2fo02377f, PMID: 36278858

[206] Wang Y Li Q Zha X Luo J . Intervention and potential mechanism of non-starch polysaccharides from natural resources on ulcerative colitis: A review. Int J Biol Macromolecules. (2022) 210:545–64. doi: 10.1016/j.ijbiomac.2022.04.208, PMID: 35513106

[207] Zheng C Chen T Lu J Wei K Tian H Liu W . Adjuvant treatment and molecular mechanism of probiotic compounds in patients with gastric cancer after gastrectomy. Food Funct. (2021) 12:6294–308. doi: 10.1039/d1fo01375k, PMID: 34052844

[208] Gaglio SC Perduca M Zipeto D Bardi G . Efficiency of chitosan nanocarriers in vaccinology for mucosal immunization. Vaccines. (2023) 11:1333. doi: 10.3390/vaccines11081333, PMID: 37631901 PMC10459455

[209] Lin W Ruishi X Caijiao X Haoming L Xuefeng H Jiyou Y . Potential applications and mechanisms of natural products in mucosal-related diseases. Front Immunol. (2025) 16:1594224. doi: 10.3389/fimmu.2025.1594224, PMID: 40370438 PMC12075308

[210] Liu W Wang S Wang J Zheng R Wang D Yu R . Neuromedin U Induces Pulmonary ILC2 Activation via the NMUR1 Pathway during Acute Respiratory Syncytial Virus Infection. Am J Respir Cell Mol Biol. (2023) 68:256–66. doi: 10.1165/rcmb.2022-0123oc, PMID: 36227802

[211] Qiu Z Xiang L Han Y Zhang B Qiao X Zheng Z . Structure-anti-inflammatory activity relationship of garlic fructans in mice with dextran sulfate sodium-induced colitis: Impact of chain length. Carbohydr Polymers. (2024) 346:122582. doi: 10.1016/j.carbpol.2024.122582, PMID: 39245481

[212] Xie S Yang G Jiang X Qin D Li Q Zha X . Polygonatum cyrtonema Hua polysaccharides promote GLP-1 secretion via activating the sweet taste receptor T1R2/T1R3-mediated cAMP signaling pathway in intestinal L cells. J Agric Food Chem. (2020) 68:6864–72. doi: 10.1021/acs.jafc.0c02058, PMID: 32456438

[213] Chen H Zhou Y Liu Y Huang J Liu H Liu C . Polysaccharides from Morus alba L. leaves alleviate knee osteoarthritis by regulating gut microbiota and inhibiting inflammation. Int J Biol Macromolecules. (2024) 269:132215. doi: 10.1016/j.ijbiomac.2024.132215, PMID: 38729482

[214] Suzuki I Itani T Ohno N Oikawa S Sato K Miyamzaki T . Polysaccharides from Grifola frondosa enhance the immune response and adjuvant activity of aluminum hydroxide in mice. J Pharmacobio-Dynamics. (1985) 8:217–26. doi: 10.1248/bpb1978.8.217, PMID: 3891963

[215] Xiao N He W Chen S Yao Y Wu N Xu M . Polysaccharides from Portulaca oleracea L. improve dextran sulfate sodium-induced colitis by regulating the NLRP3 inflammasome and gut microbiota. Mol Nutr Food Res. (2023) 68:2300509. doi: 10.1002/mnfr.202300509, PMID: 38037542

[216] Guo H Ji Z Wang B Ren J Gao W Yuan B . Polysaccharides from Lycium barbarum L. attenuate DSS-induced colitis by modulating gut microbiota and inhibiting inflammation. J Funct Foods. (2024) 119:106344. doi: 10.1016/j.jff.2024.106344

[217] Cho C Ahn S Lim T Hong H Rhee YK Yang D . Polysaccharides from Cynanchum wilfordii improve dextran sulfate sodium-induced colitis by regulating the balance of Th17/Treg cells and inhibiting inflammation. Mediators Inflammation. (2017) 2017:1–14. doi: 10.1155/2017/3859856, PMID: 28751820 PMC5496321

[218] Kanwal S Joseph TP Aliya S Song S Saleem MZ Nisar MA . Attenuation of DSS induced colitis by Dictyophora indusiata polysaccharide (DIP) via modulation of gut microbiota and inflammatory related signaling pathways. J Funct Foods. (2020) 64:103641. doi: 10.1016/j.jff.2019.103641

[219] Hao W Cha R Wang M Zhang P Jiang X . Impact of nanomaterials on the intestinal mucosal barrier and its application in treating intestinal diseases. Nanoscale Horizons. (2022) 7:6–30. doi: 10.1039/d1nh00315a, PMID: 34889349

[220] Chen Y Lin S Wang L Zhang Y Chen H Fu Z . Reinforcement of the intestinal mucosal barrier via mucus-penetrating PEGylated bacteria. Nat Biomed Engineering. (2024) 8:823–41. doi: 10.1038/s41551-024-01224-4, PMID: 38839928

[221] Wang Y Li C Li J Zhang S Zhang Q Duan J . Lycium barbarum polysaccharide fortifies intestinal mucus barrier to alleviate intestinal inflammation by modulating abundance. Acta Pharm Sin B. (2024) 14:3901–15. doi: 10.1016/j.apsb.2024.06.002, PMID: 39309495 PMC11413673

[222] Li Y Yang D Zhao H Dou L Chen Q Cheng Y . The pasteurized Weissella cibaria alleviates sepsis-induced acute lung injury by modulation of intestinal mucus barrier and gut microbiota. J Trans Med. (2025) 23:6674. doi: 10.1186/s12967-025-06674-1, PMID: 40528192 PMC12172371

[223] Yu W Luo H Han B Lin S Li Q Xue R . Restoring mucosal barrier homeostasis by in situ formation of a living-synthetic therapeutic coating. Nat Commun. (2025) 16:63110. doi: 10.1038/s41467-025-63110-0, PMID: 40825983 PMC12361522

[224] Kotla NG Isa ILM Rasala S Demir S Singh R Baby BV . Modulation of gut barrier functions in ulcerative colitis by hyaluronic acid system. Advanced Sci. (2021) 9:2103189. doi: 10.1002/advs.202103189, PMID: 34761543 PMC8811821

[225] Quan L Ouyang Y Liang W Chen Z Miao D Zheng B . Probiotic-enhanced porous bio-hybrids with inflammatory targeting, ROS scavenging, and long-term drug release for ulcerative colitis treatment. Advanced Sci. (2025) 12:2504802. doi: 10.1002/advs.202504802, PMID: 40576531 PMC12407365

[226] Cao L Peng J Duan D Cai H Cao Q Zhang W . Colon enzyme-activated prebiotic nanomedicine for targeted therapy of inflammatory bowel disease. Small Methods. (2025) 2401408. doi: 10.1002/smtd.202401408, PMID: 40509675

[227] Wang L Yan C Wang L Ai C Wang S Shen C . Lycium barbarum polysaccharide regulates gut microbiota metabolites to protect against colonic inflammation in mice. Food Funct. (2023) 14:810–21. doi: 10.1039/d2fo02964b, PMID: 36617886

[228] Chen J Gao Y Zhang Y Wang M . Research progress in the treatment of inflammatory bowel disease with natural polysaccharides and related structure-activity relationships. Food Funct. (2024) 15:5680–702. doi: 10.1039/d3fo04919a, PMID: 38738935

[229] Sun X Pei Z Wang H Zhao J Chen W Lu W . Bridging dietary polysaccharides and gut microbiome: How to achieve precision modulation for gut health promotion. Microbiological Res. (2025) 292:128046. doi: 10.1016/j.micres.2025.128046, PMID: 39793468

[230] Fang X Liu S Muhammad B Zheng M Ge X Xu Y . Gut microbiota dysbiosis contributes to α-synuclein-related pathology associated with C/EBPβ/AEP signaling activation in a mouse model of Parkinson’s disease. Neural Regeneration Res. (2024) 19:2081–8. doi: 10.4103/1673-5374.391191, PMID: 38227539 PMC11040317

[231] Xu M Shen Y Cen M Zhu Y Cheng F Tang L . Modulation of the gut microbiota-farnesoid X receptor axis improves deoxycholic acid-induced intestinal inflammation in mice. J Crohn’s Colitis. (2021) 15:1197–210. doi: 10.1093/ecco-jcc/jjab003, PMID: 33417675

[232] He X Li M Zuo X Ni H Han Y Hu Y . Kushenol I combats ulcerative colitis intestinal barrier preservation and gut microbiota optimization. World J Gastroenterol. (2025) 31:105656. doi: 10.3748/wjg.v31.i26.105656, PMID: 40678703 PMC12264850

[233] He H Dong X Cao L Sha Y Sun Y Zhao S . Oral microbiota-regulating and inflammation-targeted polymersome-hydrogels for RNAi therapy of ulcerative colitis. Bioactive Materials. (2025) 53:32–44. doi: 10.1016/j.bioactmat.2025.06.039, PMID: 40688025 PMC12272602

[234] Yang Y Zhang C Lin L Li Q Wang M Zhang Y . Multifunctional mnGA nanozymes for the treatment of ulcerative colitis by reducing intestinal inflammation and regulating the intestinal flora. ACS Appl Materials Interfaces. (2024) 16:56884–901. doi: 10.1021/acsami.4c14291, PMID: 39401179

[235] Li S Zhang Z Luo L Zhang Y Huang K Guan X . Millet quinic acid relieves colitis by regulating gut microbiota and inhibiting myD88/NF-κB signaling pathway. Foods. (2025) 14:2267. doi: 10.3390/foods14132267, PMID: 40647020 PMC12249245

[236] Kan L Zheng Z Fu W Ma Y Wang W Qian H . Recent progress on engineered micro/nanomaterials mediated modulation of gut microbiota for treating inflammatory bowel disease. J Controlled Release. (2024) 370:43–65. doi: 10.1016/j.jconrel.2024.04.014, PMID: 38608876

[237] An orally-administered nanotherapeutics with carbon monoxide supplying for inflammatory bowel disease therapy by scavenging oxidative stress and restoring gut immune homeostasis. ACS Nano. (2023)17(21):21116–33. doi: 10.1021/acsnano.3c04819.s001 37843108

[238] Crittenden S Goepp M Pollock J Robb CT Smyth DJ Zhou Y . Prostaglandin E promotes intestinal inflammation via inhibiting microbiota-dependent regulatory T cells. Sci Adv. (2021) 7:abd7954. doi: 10.1126/sciadv.abd7954, PMID: 33579710 PMC7880593

[239] Wu H Li Y Wang Y Wang Y Hong J Pang M . Anemoside B4 alleviates ulcerative colitis by attenuating intestinal oxidative stress and NLRP3 inflammasome via activating aryl hydrocarbon receptor through remodeling the gut microbiome and metabolites. Redox Biol. (2025) 85:103746. doi: 10.1016/j.redox.2025.103746, PMID: 40602277 PMC12271804

[240] Hu Y Wang Y Gao H Yang G Xie J He Z . Piperine improves DSS-induced colitis in mice via inhibition of inflammation and modulation of gut microbiota. Phytotherapy Res. (2025) 39:3197–211. doi: 10.1002/ptr.8491, PMID: 40456559

[241] Composition-activity relationships of polysaccharides from Laminaria japonica in regulating gut microbiota in short-term high-fat diet-fed mice. J Agric Food Chem. (2021) 69:11121–30. doi: 10.1021/acs.jafc.1c04490.s001, PMID: 34498470

[242] Du J Wang Z Diao X Liu Q Ma W Liu G . Bacteriocin bifidocin A from Bifidobacterium animalis subsp. animalis BB04 alleviate Listeria monocytogenes-induced intestinal infection *in vitro* and *in vivo*. Food Res Int. (2025) 214:116636. doi: 10.1016/j.foodres.2025.116636, PMID: 40467224

[243] Galacto-oligosaccharides alleviate LPS-induced immune imbalance in small intestine through regulating gut microbe composition and bile acid pool. J Agric Food Chem. (2023) 71:17615–26. doi: 10.1021/acs.jafc.3c00419.s001, PMID: 37947505

[244] Song W Wang Y Li G Xue S Zhang G Dang Y . Modulating the gut microbiota is involved in the effect of low-molecular-weight polysaccharide on immune function. Gut Microbes. (2023) 15:2276814. doi: 10.1080/19490976.2023.2276814, PMID: 37948152 PMC10653635

[245] Residue polysaccharide alleviates immunosuppression and intestinal injury by modulating gut microbiota and associated metabolites. J Agric Food Chem. (2025) 73:7788–806. doi: 10.1021/acs.jafc.4c12105.s001, PMID: 40116376

[246] Thiran A Petta I Blancke G Thorp M Plankaert G Jans M . Sterile triggers drive joint inflammation in TNF- and IL-1β-dependent mouse arthritis models. (2023). doi: 10.1101/2023.01.31.526410, PMID: 37694693 PMC10565626

[247] Zhang X Akhtar M Chen Y Ma Z Liang Y Shi D . Chicken jejunal microbiota improves growth performance by mitigating intestinal inflammation. Microbiome. (2022) 10:1299. doi: 10.1186/s40168-022-01299-8, PMID: 35836252 PMC9284917

[248] Ma Z Akhtar M Pan H Liu Q Chen Y Zhou X . Fecal microbiota transplantation improves chicken growth performance by balancing jejunal Th17/Treg cells. Microbiome. (2023) 11:1569. doi: 10.1186/s40168-023-01569-z, PMID: 37344888 PMC10283253

[249] Chen Z Liu Y Liang T Du Z Deng L Wu Z . Enhanced rotator cuff tendon-bone interface regeneration with injectable manganese-based mesoporous silica nanoparticle-loaded dual crosslinked hydrogels. Front Bioengineering Biotechnol. (2025) 13:1645970. doi: 10.3389/fbioe.2025.1645970, PMID: 40918434 PMC12409142

[250] Jin Q Lin B Lu L . Potential therapeutic value of dietary polysaccharides in cardiovascular disease: Extraction, mechanisms, applications, and challenges. Int J Biol Macromolecules. (2025) 296:139573. doi: 10.1016/j.ijbiomac.2025.139573, PMID: 39793800

[251] Liu Z Wang M Li J Liang Y Jiang K Hu Y . Hizikia fusiforme polysaccharides synergized with fecal microbiota transplantation to alleviate gut microbiota dysbiosis and intestinal inflammation. Int J Biol Macromolecules. (2024) 283:137851. doi: 10.1016/j.ijbiomac.2024.137851, PMID: 39566790

[252] Zhang L Wang S Fu X Yang Y Zhang Z Ju J . Advances in the polysaccharide derivatives for the treatment of inflammatory bowel disease: A review. Int J Biol Macromolecules. (2025) 317:144192. doi: 10.1016/j.ijbiomac.2025.144192, PMID: 40373931

[253] Yang J Lei S Wei Y Li Y Wang R Xue H . Bibliometric analysis of advances in polysaccharide pharmacology research: A review. Int J Biol Macromolecules. (2025) 313:144159. doi: 10.1016/j.ijbiomac.2025.144159, PMID: 40368214

[254] Ye Y Li M Chen W Wang H He X Liu N . Natural polysaccharides as promising reno-protective agents for the treatment of various kidney injury. Pharmacol Res. (2024) 207:107301. doi: 10.1016/j.phrs.2024.107301, PMID: 39009291

[255] Hwang J Yadav D Lee PC Jin J . Immunomodulatory effects of polysaccharides from marine algae for treating cancer, infectious disease, and inflammation. Phytotherapy Res. (2021) 36:761–77. doi: 10.1002/ptr.7348, PMID: 34962325

[256] Zhang J Zhan P Tian H . Recent updates in the polysaccharides-based Nano-biocarriers for drugs delivery and its application in diseases treatment: A review. Int J Biol Macromolecules. (2021) 182:115–28. doi: 10.1016/j.ijbiomac.2021.04.009, PMID: 33836188

[257] Maryam S Krukiewicz K . Sweeten the pill: Multi-faceted polysaccharide-based carriers for colorectal cancer treatment. Int J Biol Macromolecules. (2024) 282:136696. doi: 10.1016/j.ijbiomac.2024.136696, PMID: 39437958

[258] Guo H Wang N Li S Li Z . The roles of polysaccharides in rheumatoid arthritis treatment: A review focusing on the sources, structures and their related mechanism. Int J Biol Macromolecules. (2025) 321:146377. doi: 10.1016/j.ijbiomac.2025.146377, PMID: 40738445

[259] Bai X Feng Z Peng S Zhu T Jiao L Mao N . Chitosan-modified Phellinus igniarius polysaccharide PLGA nanoparticles ameliorated inflammatory bowel disease. Biomaterials Advances. (2022) 139:213002. doi: 10.1016/j.bioadv.2022.213002, PMID: 35882149

[260] Wang Z Fu S Guo Y Han Y Ma C Li R . Classification and design strategies of polysaccharide-based nano-nutrient delivery systems for enhanced bioactivity and targeted delivery: A review. Int J Biol Macromolecules. (2024) 256:128440. doi: 10.1016/j.ijbiomac.2023.128440, PMID: 38016614

[261] Zang J Kou Y Shi Y Xiao L Ma K Zhang C . Structural and functional roles of lactic acid bacteria in food delivery systems: A dual perspective of passive encapsulation and active carriers. Adv Colloid Interface Science. (2025) 344:103599. doi: 10.1016/j.cis.2025.103599, PMID: 40690838

[262] Alavi M Ashengroph M . Micro- and nanoformulations of functionalized biopolymers in neurodegenerative diseases, diabetes, cancer, microbial infections, and regenerative medicine: A comprehensive review. Int J Biol Macromolecules. (2025) 322:146729. doi: 10.1016/j.ijbiomac.2025.146729, PMID: 40816380

[263] Dessai AD Nair A Selvasudha N Garg S Nayak UY . Glyco-nanomaterials as a therapeutic target in drug delivery and biomedicine: A review. Int J Biol Macromolecules. (2025) 329:147830. doi: 10.1016/j.ijbiomac.2025.147830, PMID: 40987360

[264] Huang L Luo S Tong S Lv Z Wu J . The development of nanocarriers for natural products. WIREs Nanomedicine Nanobiotechnol. (2024) 16:1967. doi: 10.1002/wnan.1967, PMID: 38757428

[265] Fatima G Khan S Shukla V Awaida W Li D Gushchina YS . Nutraceutical formulations and natural compounds for the management of chronic diseases. Front Nutr. (2025) 12:1682590. doi: 10.3389/fnut.2025.1682590, PMID: 41170369 PMC12568601

[266] Hong CR Lee EH Jung YH Lee J Paik H Hong S . Development and characterization of extract-loaded liposomes: potential as anti-inflammatory functional food ingredients. Antioxidants. (2023) 12:1636. doi: 10.3390/antiox12081636, PMID: 37627631 PMC10451523

[267] Bian Z Zhao A Wang Q Li Y Liu Y Yang W . Advancements in research on the anti-metabolic dysfunction-associated steatotic liver disease effects and mechanisms of action of traditional Chinese medicine polysaccharides: A review. Int J Biol Macromolecules. (2025) 321:146292. doi: 10.1016/j.ijbiomac.2025.146292, PMID: 40716550

[268] Li X Zhang X Liu L Feng L Liu B . Polysaccharide-based nanosystems as vaccine adjuvants: A review. Carbohydr Polymers. (2025) 367:124017. doi: 10.1016/j.carbpol.2025.124017, PMID: 40817549

[269] Bamigbade GB Abdin M Subhash A Arachchi MP Ullah N Gan R . Plant polysaccharide-capped nanoparticles: A sustainable approach to modulate gut microbiota and advance functional food applications. Compr Rev Food Sci Food Saf. (2025) 24:70156. doi: 10.1111/1541-4337.70156, PMID: 40052474 PMC11887029

[270] Lai Y Zhang Q Xu M Guo X Zhou Q Liu Q . Restoration of antibiotic associated diarrhea induced gut microbiota disorder by using water-insoluble polysaccharides in C57BL/6J mice. Front Nutr. (2025) 12:1607365. doi: 10.3389/fnut.2025.1607365, PMID: 40709339 PMC12286944

[271] Wang X Wang J Bi H Zhang Z Zhang M Wang M . Research progress in the extraction and purification, structural characteristics, pharmacological activities, structure-activity relationships, and applications from Alpinia oxyphylla Miq. polysaccharides. Int J Biol Macromolecules. (2025) 315:144387. doi: 10.1016/j.ijbiomac.2025.144387, PMID: 40409621

[272] Polysaccharides enhance the preventive efficacy of heat-inactivated WX-94 against high-fat-high-sucrose-induced liver injury and gut dysbacteriosis. J Agric Food Chem. (2024) 72(17):9880–92. doi: 10.1021/acs.jafc.4c00372.s001, PMID: 38646869

[273] Chen P Chen X Hao L Du P Li C Han H . The bioavailability of soybean polysaccharides and their metabolites on gut microbiota in the simulator of the human intestinal microbial ecosystem (SHIME). Food Chem. (2021) 362:130233. doi: 10.1016/j.foodchem.2021.130233, PMID: 34090043

