# Correction: Anti-inflammatory effects of natural polysaccharides: molecular mechanisms and nanotherapeutic applications

**DOI:** 10.3389/fimmu.2026.1797602

**Published:** 2026-02-02

**Authors:** Frontiers Production Office

**Affiliations:** Frontiers Media SA, Lausanne, Switzerland

**Keywords:** natural polysaccharides, anti-inflammation, molecular mechanism, nanonization, therapeutic application

Affiliation “Department of Gastroenterology, The Second Affiliated Hospital, Jiangxi Medical College, Nanchang University, Jiangxi, China” was erroneously omitted for author “Jihao Yang”.

The original version of this article has been updated.

